# The SUN Protein Mps3 Is Required for Spindle Pole Body Insertion into the Nuclear Membrane and Nuclear Envelope Homeostasis

**DOI:** 10.1371/journal.pgen.1002365

**Published:** 2011-11-17

**Authors:** Jennifer M. Friederichs, Suman Ghosh, Christine J. Smoyer, Scott McCroskey, Brandon D. Miller, Kyle J. Weaver, Kym M. Delventhal, Jay Unruh, Brian D. Slaughter, Sue L. Jaspersen

**Affiliations:** 1 Stowers Institute for Medical Research, Kansas City, Missouri, United States of America; 2 Department of Molecular and Integrative Physiology, University of Kansas Medical Center, Kansas City, Kansas, United States of America; Duke University, United States of America

## Abstract

The budding yeast spindle pole body (SPB) is anchored in the nuclear envelope so that it can simultaneously nucleate both nuclear and cytoplasmic microtubules. During SPB duplication, the newly formed SPB is inserted into the nuclear membrane. The mechanism of SPB insertion is poorly understood but likely involves the action of integral membrane proteins to mediate changes in the nuclear envelope itself, such as fusion of the inner and outer nuclear membranes. Analysis of the functional domains of the budding yeast SUN protein and SPB component Mps3 revealed that most regions are not essential for growth or SPB duplication under wild-type conditions. However, a novel dominant allele in the P-loop region, *MPS3-G186K*, displays defects in multiple steps in SPB duplication, including SPB insertion, indicating a previously unknown role for Mps3 in this step of SPB assembly. Characterization of the *MPS3-G186K* mutant by electron microscopy revealed severe over-proliferation of the inner nuclear membrane, which could be rescued by altering the characteristics of the nuclear envelope using both chemical and genetic methods. Lipid profiling revealed that cells lacking *MPS3* contain abnormal amounts of certain types of polar and neutral lipids, and deletion or mutation of *MPS3* can suppress growth defects associated with inhibition of sterol biosynthesis, suggesting that Mps3 directly affects lipid homeostasis. Therefore, we propose that Mps3 facilitates insertion of SPBs in the nuclear membrane by modulating nuclear envelope composition.

## Author Summary

Accurate segregation of chromosomes during mitosis is essential to prevent genetic instability and aneuploidy that lead to cancer and other diseases. Centrosomes and spindle pole bodies mediate the assembly of a microtubule-based structure known as the mitotic spindle, which physically separates chromosomes during mitosis so that the two daughter cells contain a complete copy of the genetic material as well as a spindle pole. During every cell cycle, the DNA and the spindle pole must be duplicated exactly once to ensure proper formation of a bipolar mitotic spindle. In yeast cells, the nuclear envelope does not break down, so the spindle pole must be inserted into the nuclear membrane so that it can form both the microtubules involved in the mitotic spindle and those involved in positioning of the nucleus. How a large protein complex such as the spindle pole body is inserted into the lipid layers of the nuclear membrane is not well understood. We show that the evolutionarily conserved SUN protein Mps3 is involved in spindle pole insertion into the nuclear membrane. This likely reflects a function for SUN proteins in controlling nuclear envelope structure by modulating the types of lipids that are present in the nuclear membrane.

## Introduction

The hallmark feature of eukaryotic cells is the nucleus, a double membrane bound organelle that contains the genetic material. The outer nuclear membrane (ONM) of the nucleus is contiguous with the ER membrane while the inner nuclear membrane (INM) is distinct and contains a unique set of proteins that interact with chromatin and other nuclear factors. Embedded in the nuclear membrane are multiple nuclear pore complexes (NPCs) that regulate transport of macromolecules between the cytoplasm and the nucleus [1]. In organisms such as *Saccharomyces cerevisiae* that undergo a closed mitosis, the centrosome-equivalent organelle known as the spindle pole body (SPB) is present in the nuclear envelope throughout the life cycle [2]. The SPB organizes both cytoplasmic microtubules, which are involved in nuclear positioning, and nuclear microtubules, which are essential for chromosome segregation [3].

Both NPCs and SPBs are composed primarily of soluble proteins that partially assemble into sub-complexes in the nucleus or cytoplasm (reviewed in [1], [3]). Further assembly of both NPCs and SPBs requires insertion into the nuclear membrane at a point where the INM and ONM are joined together. Specific integral membrane proteins interact with soluble components of the NPC and SPB and are thought to anchor the complexes in the nuclear envelope. Ndc1 is essential for insertion of both the NPC and SPB [4]–[6]. At the NPC, three additional pore membrane proteins, Pom33, Pom34 and Pom152, play partially overlapping roles in NPC assembly [6]–[8], while Nbp1, Bbp1 and Mps2 are required in addition to Ndc1 for SPB insertion into the nuclear envelope [9]–[12].

The mechanism of NPC insertion has been extensively studied in both yeast and metazoan systems. Structural studies have shown that five subunits of the NPC (Nup133, Nup120, Nup85, Nup170 and Nup188) contain an ALPS motif (for ArfGAP1 lipid packing sensor), which targets them to highly curved membranes [13]. These proteins are thought to form a coat complex on the nuclear envelope to facilitate NPC insertion [14]–[17]. In addition, membrane-bending proteins of the ER such as the reticulons have been shown to play a role in *de novo* NPC assembly [17], [18]. Modification of lipids within nuclear membrane leaflets probably also occur at sites of NPC insertion to accommodate membrane curvature and fusion. Several proteins involved in lipid synthesis and membrane fluidity have been genetically linked to NPC assembly [19]–[21], although their role in NPC insertion is not well characterized. In vertebrates cells, the SUN (for Sad1-UNC-84 homology) protein Sun1 also is required for NPC assembly [22], [23]. A recent study suggested that hSun1 together with Pom121 is required for *de novo* assembly of NPCs possibly by facilitating membrane fusion [24].

The mechanism of SPB insertion into the nuclear membrane is poorly understood in comparison to insertion of NPCs. It is possible that many of the same events, such as membrane bending, curvature and lipid modification, are needed for SPB duplication since fusion of INM and ONM also must occur during SPB insertion. No specific factors that possess these functions have ever been directly implicated in the SPB duplication process with the exception of a recent report suggesting that the amphipathic alpha-helix of Nbp1 aids SPB insertion [12]. Perhaps one of the best clues as to how the SPB might insert into the nuclear membrane comes from a plethora of genetic interactions that have been identified between genes encoding SPB components and NPC subunits, including suppression of complete deletions of the SPB membrane components, *MPS2* and *MPS3*, by *POM34* or *POM152* deletion [4], [25]–[27]. While it is possible that the NPC is involved in SPB insertion through its role in nuclear translocation of SPB subunits or in mRNA processing [25], [28], the fact that SPB duplication can occur in the absence of certain structural subunits points to a model in which NPCs and SPBs compete for a shared insertion factor, such as Ndc1 [4]. Alternatively, blocking NPC assembly by elimination of *POM152* or *POM34* could alter some aspect of the nuclear membrane that enables the SPB to duplicate in the absence of otherwise essential components. Consistent with this possibility, deletion of *POM152* together with *NUP170* results in nuclear membrane abnormalities [29].

In the present study, we further examine the role of the nuclear membrane in SPB duplication and show for the first time that changes in membrane composition are sufficient for SPB duplication in the absence of Mps3. In addition, we demonstrate a role for the SUN protein Mps3 in regulation of membrane architecture and provide evidence that it functions in the insertion step of SPB duplication. This role of Mps3 is distinct from the previously described functions of Mps3 in SPB duplication. As a component of the SPB substructure that templates assembly for the new SPB, known as the half-bridge, Mps3 is required for initiation of SPB duplication and for tethering the half-bridge to the soluble core SPB through its interaction with the membrane protein Mps2 [30]–[32]. This function is similar to that of other SUN domain-containing proteins, which have been shown to play a role in accurate chromosome segregation and nuclear positioning due to their role in duplication and membrane tethering of centrosomes, basal bodies and SPBs in a wide variety of systems [33]–[35]. However, our current data suggest that SUN proteins have a novel function in membrane homeostasis, which is involved in insertion of protein complexes such as the SPB into the nuclear envelope.

## Results

### Identification of Mps3 domains that are required for SPB duplication

**Figure 1:**
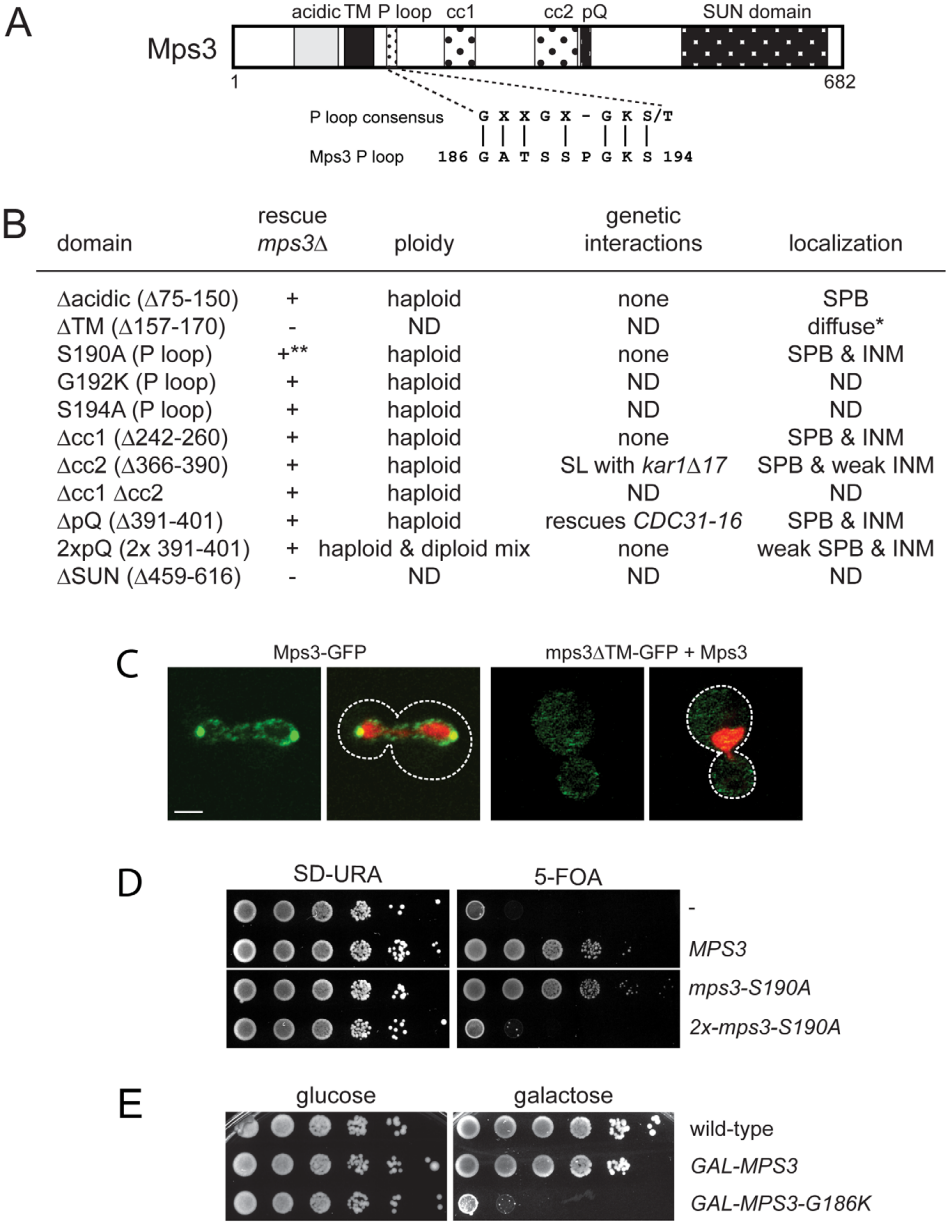
Essential regions of Mps3 for SPB duplication. (A) Schematic of Mps3 showing the N-terminal acidic region, transmembrane domain (TM), P-loop motif, coiled-coils (cc), poly-glutamine (pQ) repeat and C-terminal SUN domain. Alignment of P-loop consensus sequence with amino acids 186–194 of Mps3 is shown below. (B) The indicated mutants within each Mps3 domain were tested for their ability to rescue a deletion of *MPS3* (SLJ2039). + and − indicate growth and death, respectively, of mutants on 5-fluoroorotic acid (5-FOA) at 30°C. Viable alleles were analyzed further to determine DNA content by flow cytometry and tested for genetic interactions with other SPB mutants, including *kar1Δ17* (SLJ844), *cdc31-2* (SLJ894), *mps2-1* (SLJ718), *sfi1-3* (SLJ1558) and *CDC31-16* (SLJ907). Alleles were also fused to GFP to determine the subcellular distribution of the mutant protein using confocal imaging [41]. *Non-functional mutants were localized in the presence of wild-type Mps3. ND, not determined. **The *mps3-S190A* is dependent of copy number: single copy integrants are viable while multiple copy integrants are lethal, as shown in the serial dilution assay in (D). (C) A representative single plane confocal image showing the localization of Mps3-GFP (SLJ2571) and mps3ΔTM-GFP (SLJ4308) (green) together with H2B-mCherry (red) in cells grown at 23°C. Bar, 2 µm. The covering plasmid was removed from SLJ2571 by plating cells to 2-amino-5-fluorobenzoic acid (5-FBA) plates immediately prior to use; because mps3ΔTM-GFP is non-functional, the wild-type untagged copy of *MPS3* could not be lost. (D) Serial dilution assay of SLJ2039 containing no insert (-), wild-type *MPS3*, or *mps3-S190A* integrated in 1 copy or 2 copies on SD-URA and 5-FOA, which removes the covering plasmid containing wild-type *MPS3*. Plates were grown for 2 d at 30°C. (E) Wild-type (SLJ001), *GAL-MPS3* (SLJ995) and *GAL-MPS3-G186K* (SLJ1797) cells were tested for their ability to grow on YPD (glucose) and YPGR (galactose) plates at 30°C in a serial dilution assay.

A key feature of all SUN proteins, including Mps3, is the presence of a number of structural motifs, including at least one transmembrane domain, regions of coiled-coils and a C-terminal SUN domain [35]. In addition, Mps3 contains an N-terminal acidic domain, a poly-glutamine region and a putative P-loop [30] (Figure 1A). In order to determine their role in SUN protein localization and function, we created deletion alleles in each domain using site directed mutagenesis. In the case of the poly-glutamine repeat (pQ), we not only deleted this region but also expanded it two-fold since this type of mutation is often linked to disease [36], [37]. In the case of the putative P-loop, we mutated key residues anticipated to be involved in nucleotide binding to a residue with different chemical properties [38], [39]. To test if the various mutants were functional, we assayed complementation of an *mps3Δ* mutant using a plasmid shuffle strategy. Although Mps3 plays multiple non-essential roles in the chromosome organization within the nucleus [28], [40]–[49], the primary function of Mps3 in mitotically dividing yeast cells is at the SPB [30], [31], so this test will allow us to identify domains of Mps3 that are essential for SPB duplication.

*mps3Δcc1*, *mps3Δcc2*, *mps3Δcc1Δcc2* and *mps3ΔpQ* mutants, which lack the first, second and both coiled-coil domains or the poly-glutamine region, respectively, did not exhibit any obvious growth defect at various temperatures (Figure 1B; data not shown). To further examine their role in Mps3 function at the SPB, we tested for genetic interactions with mutants in other SPB components: *mps2-1*, *cdc31-2*, *CDC31-16*, *kar1Δ17*, *spc42-11*, *spc29-3* and *sfi1-3*. Each of these mutants is either inviable (synthetic lethal) or shows an enhanced growth defect (synthetic sick) with *mps3-1*, which has serine 472 in the SUN domain mutated to asparagine [30]. Like *MPS3*, *KAR1* and *CDC31* encode components of the SPB half-bridge that are required for the initial step of SPB duplication: Kar1 is an integral membrane protein and Cdc31 is a small calcium binding protein that also binds to Sfi1 on the cytoplasmic side of the SPB half-bridge. Spc42 and Spc29 are components of the core SPB and Mps2 is a linker protein that tethers the half-bridge to the core SPB through interactions with Mps3 [3], [32]. Surprisingly, only two genetic interactions were discovered with this new panel of *mps3* alleles: synthetic lethality between *mps3Δcc2* and *kar1Δ17*, and suppression of the temperature sensitivity of the dominant *CDC31-16* mutant by *mps3ΔpQ* (Figure 1B). However, unlike *mps3-1* mutants [30], Cdc31 and Kar1 localization to the SPB was unaffected in *mps3Δcc2* or *mps3ΔpQ* mutants (data not shown). Most likely, this is because mps3Δcc1-GFP, mps3Δcc2-GFP and mps3ΔpQ-GFP localize to the SPB and INM in a pattern that is highly similar to that of Mps3-GFP (Figure 1B). Taken together, our data suggests that mutation of the coiled-coil domains or deletion of the poly-glutamine region have at most minor effects on the localization and function of Mps3 during SPB duplication.

Duplication of the poly-glutamine region did not affect cell growth (Figure 1B). However, analysis of DNA content by flow cytometry revealed that *mps3-2xpQ* mutants exhibited an increase in ploidy (Figure 1B), which is a common phenotype in SPB mutants, and it has been previously observed in a number of *mps3* alleles [30], [32], [40]. The increase in ploidy was fully recessive as cells containing *mps3-2xpQ* and a wild-type copy of *MPS3* were haploid (data not shown), indicating that although the mps3-2xpQ protein may be only partially functional, it does not form a complex that titrates out other SPB duplication factors. Our observation that mps3-2xpQ-GFP levels at the SPB are reduced compared to Mps3-GFP (Figure 1B) suggests that this mutant is unable to localize to the SPB, perhaps due to a change in binding with its receptor at the SPB.

Similar to mutants that eliminate or affect the SUN domain [30]–[32], deletion of the transmembrane domain resulted in a non-functional version of Mps3 (Figure 1B). This is most likely due to mislocalization of the mutant protein since mps3ΔTM-GFP was only visible in a diffuse pattern throughout the cytoplasm and the nucleus even in the presence of a wild-type untagged copy of Mps3 rather than at both SPBs and the peripheral nuclear envelope like Mps3-GFP (Figure 1B) [41]. Replacement of the Mps3 transmembrane domain with that of several other membrane proteins rescued the lethality of *mps3Δ* and restored localization to the SPB and the peripheral nuclear envelope (Figure S1A and S1B). Interestingly, some of these chimeric proteins displayed significantly different localization patterns from wild-type Mps3-GFP (Figure S1A). The fact that these proteins were sufficient to target enough Mps3 to the SPB to allow for cell proliferation and maintenance of genomic stability indicates that although membrane localization is critical for Mps3 function during SPB duplication, a specific transmembrane domain sequence is not required to target the SUN protein to the SPB.

### Mutation of P-loop residues results in a novel dominant *MPS3* allele

When point mutants were constructed in potential residues involved in nucleotide binding within the P-loop region, we found most alleles were able to complement *mps3Δ* and serve as the sole copy of *MPS3* in the cell (Figure 1B; data not shown), suggesting that this domain does not function in ATP-binding *in vivo*. However, some mutants in the P-loop region such as *mps3-S190A* displayed copy number sensitivity such that cells containing a single integrated copy of the mutant gene were viable, but cells containing two or more copies of the P-loop mutant gene were dead (Figure 1D). This indicates that *mps3-S190A* is a weak dosage-sensitive antimorphic allele. We were unable to obtain transformants of one allele (*MPS3-G186K*) under a wide variety of conditions (data not shown), suggesting that it is a dominant mutant that arrests cell growth.

Using the galactose-regulatable *GAL1-10* promoter, we set up a system so that we could examine the effects of *MPS3-G186K* on cell growth and SPB duplication. Cells containing a single integrated copy of *GAL-MPS3* or *GAL-MPS3-G186K* in addition to the endogenous wild-type copy of *MPS3* were analyzed for their effect on growth in a serial dilution assay at 30°C. Under these conditions, overexpression of wild-type *MPS3* had a slight effect on cell growth while overexpression of *MPS3-G186K* significantly inhibited cell proliferation (Figure 1E). This confirms that *MPS3-G186K* is a novel dominant lethal mutant.

### *MPS3-G186K* mutants are defective in SPB duplication

**Figure 2:**
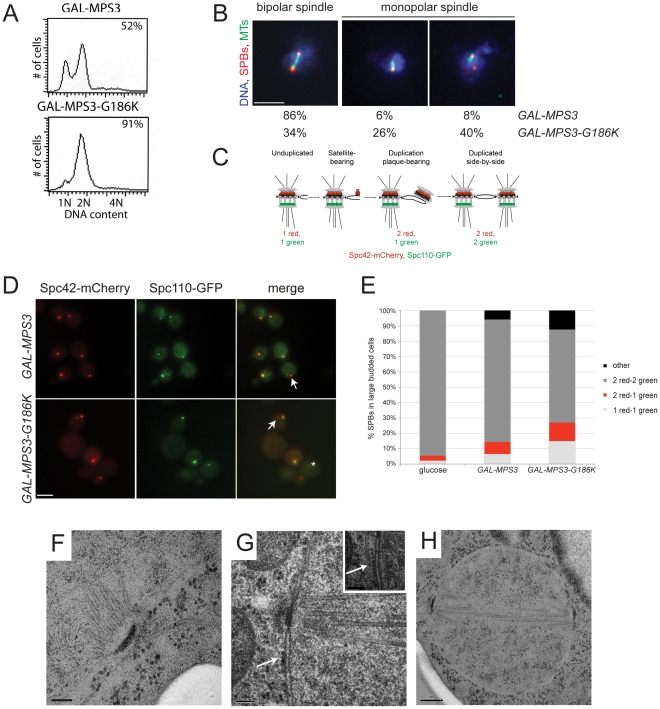
*MPS3-G186K* expression causes a mitotic arrest due to SPB duplication defects. (A,B) *GAL-MPS3* (SLJ995) and *GAL-MPS3-G186K* (SLJ1797) cells were grown overnight at 30°C in YEP plus 2% raffinose then 2% galactose was added for 5 h to induce expression. (A) DNA content was analyzed by flow cytometry and budding index was determined using phase contrast microscopy. The percentage of large-budded cells is indicated (n = 300). (B) In addition, spindle morphology was examined using indirect immunofluorescence microscopy using anti-α-tubulin (green), anti-γ-tubulin (red) and DAPI (blue). The percentage of large budded cells (n>200) with bipolar and both types of monopolar spindles is indicated for each strain. Bar, 5 µm. (C) Schematic of SPB duplication pathway showing the timing of Spc110-GFP (green) and Spc42-mCherry (red) assembly. (D) SPBs were visualized using Spc110-GFP (green) and Spc42-mCherry (red) in *GAL-MPS3* (SLJ4859) and *GAL-MPS3-G186K* (SLJ4864) cells grown for 5 h in SC-HIS+2% galactose+2% raffinose at 30°C. Arrows point to unduplicated SPBs that contain only Spc42-mCherry, and an asterisk marks a SPB fragment seen in *MPS3-G186K*, which contains only Spc110-GFP. This may represent a delaminated SPB similar to that observed in some *spc110* mutants [101]. Bar, 5 µm. (E) The number of SPBs containing the indicated number of Spc42-mCherry and Spc110-GFP foci in large budded cells was quantitated in two independent experiments (n>250). We also counted SPB foci in SLJ4859 cells grown in SC-HIS+2% raffinose+2% glucose for 5 h at 30°C. (F–H) *GAL-MPS3-G186K* (SLJ1797) mutants were also processed for thin-section EM to examine SPB morphology following induction for 5 h in YPGR at 30°C. Serial sections through nuclei of 15 cells were collected: (F) 7 nuclei contained a single unduplicated SPB, (G) 4 nuclei contained an SPB as well as electron dense material (arrow) resembling a SPB precursor on the cytoplasmic face of the nuclear envelope, and (H) 4 nuclei contained duplicated and separated SPBs, which nucleated a short bipolar spindle. The inset in (G) shows an adjacent section with a cytoplasmic microtubule emanating from the SPB precursor (position marked with an arrow). (F,G) Bar, 100 nm. (H) Bar, 200 nm.

These same strains were examined following a 5 h induction with 2% galactose by flow cytometry to analyze DNA content and by indirect immunofluorescence microscopy with anti-alpha-tubulin and anti-gamma-tubulin antibodies to visualize microtubules and SPBs, respectively, to determine if the *MPS3-G186K* mutant affected SPB duplication and spindle assembly. We found that cells overproducing wild-type *MPS3* did not arrest and underwent SPB duplication to form bipolar mitotic spindles (Figure 2A, 2B). However, *MPS3-G186K* overproduction resulted in an accumulation of large-budded cells with a 2N DNA content, which is suggestive of a failure in SPB duplication and/or spindle formation (Figure 2A). Examination of microtubule structures showed that 66% of *MPS3-G186K* mutants, but only 14% of *GAL-MPS3* cells, contain monopolar spindles: a single DNA mass associated with a single SPB and microtubule array (Figure 2B; n>200). Unlike the monopolar spindle phenotype previously described for *mps3* mutants where a single focus of gamma-tubulin is associated with the nuclear DNA [30], [32], 40% of *MPS3-G186K* cells contain two foci of gamma-tubulin, one of which does not nucleate microtubules (Figure 2B). This phenotype is indicative of a defect in SPB insertion into the nuclear envelope: the SPB that does not nucleate microtubules has not properly inserted into the nuclear envelope and assembled inner plaque components, which are necessary for the formation of a nuclear microtubule array [3]. Although the uninserted SPB may nucleate cytoplasmic microtubules, these are generally difficult to observe due to the small number that is formed at each SPB. This phenotype is highly reminiscent of that observed in mutants that are defective in SPB insertion into the nuclear envelope such as *mps2-1*, *ndc1-1*, *nbp1-dg* and *bbp1-1* [9]–[11], [50] and suggests that like these SPB components, Mps3 has a function in the late step of SPB duplication in addition to its role in initiation of SPB duplication.

### *MPS3-G186K* mutants are defective in SPB insertion

In order for inner plaque components of the SPB such as Spc110 to assemble onto the newly duplicated SPB, the new SPB must be inserted into the nuclear envelope (Figure 2C). Cells defective in SPB insertion due to a mutation in *MPS2* or *BBP1* contain two foci of the fluorescently-labeled central plaque component Spc42, which is present at the old SPB and the duplication plaque/new SPB, but a single focus of fluorescently-labeled Spc110 [11], [25]. In *GAL-MPS3* cells, we found that in 80% of cells containing two Spc42-mCherry foci, those foci were coincident with two Spc110-GFP foci, indicating that these SPBs had duplicated and inserted into the nuclear envelope (Figure 2D–2E). In contrast, only 61% of *GAL-MPS3-G186K* cells contained two SPBs that were labeled with both Spc42-mCherry and Spc110-GFP. 15% of the remaining cells contained a single SPB with both Spc42-mCherry and Spc110-GFP, indicative of an unduplicated SPB, 12% of cells contained two SPBs labeled with Spc42-mCherry only one of which co-labeled Spc110-GFP, indicative of an arrest at an intermediate step in SPB duplication, and 12% contained multiple SPB foci (Figure 2D–2E). Thus, *MPS3-G186K* has pleiotropic effects on SPB duplication, including blocking SPB insertion into the nuclear envelope. Based on the fact that virtually all wild-type cells have two duplicated SPBs that co-labeled with Spc42-mCherry and Spc110-GFP, the increased frequency in aberrant pole morphologies that we observed in *MPS3-G186K* is striking and highly statistically significant (p<0.01). In addition, the levels of uninserted poles in *MPS3-G186K* is similar to that observed in other mutants such as *mps2-1* and *ndb1-dg*, which fail in the late step of SPB duplication (see [25]).

We used electron microscopy (EM) to further evaluate the SPB duplication defect of cells overexpressing *MPS3-G186K*. Serial section analysis through the entire nucleus revealed that 11 out of 15 nuclei examined contained a single SPB. Of these 11 monopolar spindles, 4 had evidence of a “dead” pole characteristic of mutants defective in the late step of SPB insertion [9]–[11], [50]; that is, an electron dense structure associated with the nucleus and with cytoplasmic microtubules but not inserted into the nuclear envelope (Figure 2G). The remaining 7 SPBs appeared to be unduplicated, arresting with a terminal morphology similar to previously described *mps3* alleles (Figure 2F) [30], [32]. The remaining 4 cells contained short bipolar spindles (Figure 2H). This ultrastructural analysis is consistent with our fluorescence and genetic data and suggests that *MPS3-G186K* affects multiple steps in SPB duplication, including SPB insertion into the nuclear envelope.

We tested the ability of overexpressed SPB components to suppress toxicity of the mutant on galactose and found that 2μ-*MPS3* partially rescued growth, confirming that *MPS3-G186K* is an antimorphic allele (Figure S2). Most other SPB components failed to rescue the growth arrest. The notable exception was 2μ-*SPC42*. Spc42 is a component of the SPB central plaque that serves as a scaffold for assembly of the organelle and plays a role in anchorage in the nuclear membrane [51], [52]. The fact that its overexpression partially restores growth to *MPS3-G186K* mutants is consistent with a defect in membrane insertion and tethering.

### *MPS3-G186K* cells have a membrane proliferation defect

**Figure 3:**
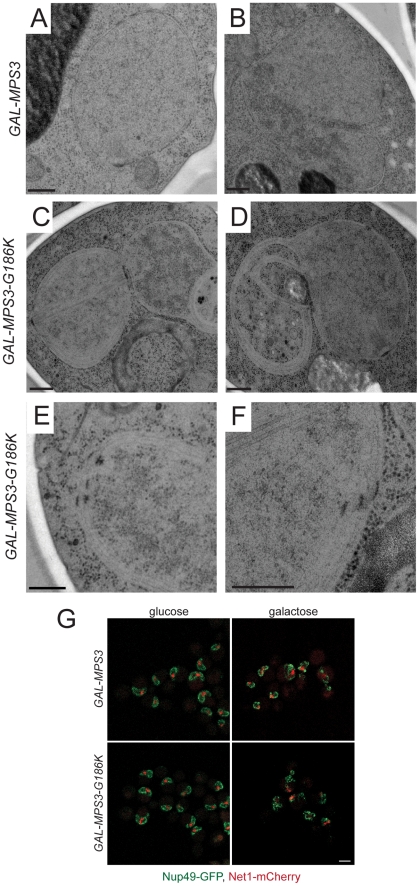
Over-proliferation of the nuclear membrane in *GAL-MPS3-G186K*. *GAL-MPS3* (SLJ995) and *GAL-MPS3-G186K* (SLJ1797) cells were processed for thin-section EM following induction for 5 h in YPGR at 30°C. (A) Nuclei from *GAL-MPS3* cells show a round or elliptical morphology (50%; n = 17) similar to that seen in wild-type cells (see [55]), or (B) an abnormal nuclear morphology (50%; n = 17). In all nuclei from *GAL-MPS3* cells, only a single nuclear envelope bi-layer was observed. (C–F) Nuclei from *GAL-MPS3-G186K* all (n = 50) exhibited an abnormal nuclear morphology, which included multiple lobes (C), as well as highly curved projections (D). Multiple nuclear envelope layers were present, and this often resulted in (E) clustered NPCs in regions containing a single nuclear membrane layer. In other cases (F), stacked NPCs were observed. (A–F) Bar, 200 nm. (G) *GAL-MPS3* (SLJ5097) and *GAL-MPS3-G186K* (SLJ5098) cells containing Nup49-GFP and Net1-mCherry were grown to mid-log phase in YEP+2% raffinose, then 2% glucose was added to half and 2% galactose was added to the other half and cells were allowed to grow for 5 h at 30°C. Mislocalization of Nup49-GFP (green) and Net1-mCherry (red) in *MPS3-G186K* in galactose are consistent with aberrant nuclear morphology seen by EM, but no NPCs were observed in the cytoplasm in either mutant. Bar, 5 µm.

In addition to the SPB duplication defect, several additional phenotypes were uncovered during the course of our EM analysis of *MPS3-G186K* cells that shed light onto the possible function of Mps3 in SPB insertion. As depicted in Figure 3C–3F, *MPS3-G186K* mutants appeared to have undergone massive over-proliferation of the nuclear membrane, resulting in 2–8 layers of nuclear envelope, and nuclei appear to have multiple lobes and extensions. The membrane expansion phenotype was highly specific and penetrant, occurring in 96% (48 of 50) of *GAL-MPS3-G186K* cells examined (Figure 3C–3F) but in none (0 of 34) of the *GAL-MPS3* cells (Figure 3A–3B). Therefore, it is most probably due to an effect of the *MPS3-G186K* mutant and not a general result due to overexpression of *MPS3* or an integral membrane protein (see also [31]). The fact that we observe excess membrane in *MPS3-G186K* mutant suggests that Mps3 directly or indirectly is involved in membrane homeostasis.

**Figure 4:**
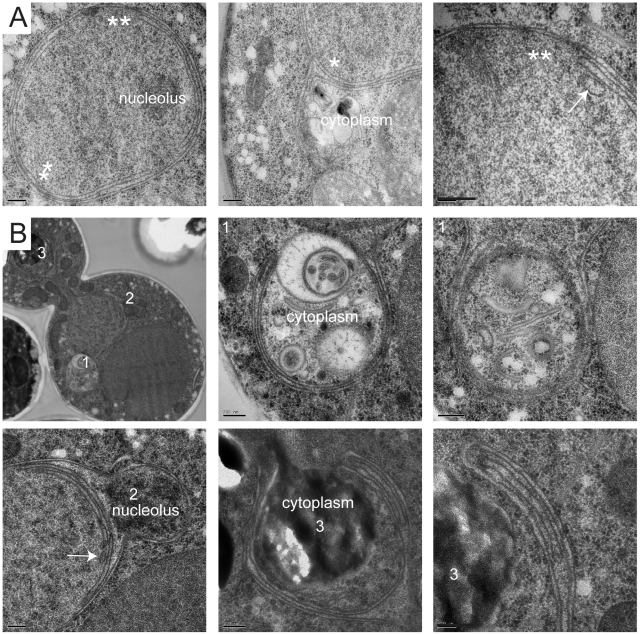
Formation of tubules and intranuclear membranes in *MPS3-G186K* cells. EM images from *GAL-MPS3-G186K* cells grown for 5 h in YPGR at 30°C showing the formation of intranuclear tubules. (A) In the cell on the left, the positions of tubule formation are indicated by a single or double asterisk, and higher magnification images of these regions are shown on the right. In the center image, the outer layers of the nuclear membrane appear to have extended into the cytoplasm to enclose a region of the cytosol. An arrow points to a tubule forming what appears to be a third nuclear layer in the right image. (B) The cell shown has at least three interesting regions of membrane expansion, which are denoted and shown in higher magnification. In region 1, at least two lipid bilayers have encircled a region of the cytosol. In region 2, expansion of membranes within the nucleus is not uniform. Here, membrane proliferation is occurring inside the nucleus to compartmentalize the nucleolus away from rest of the nucleus. In region 3, membrane is also encircling a cytoplasmic region, but it is clear that the membranes are folding back on themselves to form a tubule inside the membrane bilayers. Bars, 200 nm.

Membrane proliferation in *GAL-MPS3-G186K* was restricted to the nucleus; no excess membrane was seen on other organelles, including the ER that is contiguous with the ONM in budding yeast (Figure 4). Previous work had suggested that the membrane adjacent to the nucleolus was subject to the formation of membrane flares [53], but we found that all areas of the nuclear membrane underwent expansion, not just the nucleolar membrane. However, we did observe that the nucleolar region was often partitioned away from the main mass of the nucleus either by a membrane (Figure 4B) or by the formation of a lobe (Figure 3C). Interestingly, within an individual nucleus, membrane proliferation was not uniform in that there were regions that contained a single bilayer and other regions containing multiple bilayers (Figure 3C and 3D, Figure 4). Analysis of serial nuclear sections showed that the excess membrane begins as tubules within the nucleus, which then proliferate underneath the existing membrane and then fuse with adjacent tubules (Figure 4A) or fold back upon itself to continue proliferation within the nucleus (Figure 4B). The fact that the excess membrane forms only in tight association with existing nuclear envelope and does not form lamellae within the nucleus suggests that Mps3 is intimately involved in formation of the nuclear envelope layers.

While it is possible that the excess membrane in *MPS3-G186K* inhibits SPB insertion, we found that SPBs as well as NPCs were often associated with a membrane region containing a single bilayer (Figure 2F–2H and Figure 3C, 3E). However, we also detected NPCs in intermediate and inner layers, although these often appeared to be stacked with NPCs in adjacent layers (Figure 3F), most likely to facilitate nuclear-cytoplasmic trafficking of macromolecules, which is reduced but not completely inhibited in *MPS3-G186K* (Figure S3). Unlike other mutants that affect the yeast nuclear membrane [18]–[21], [54], we did not observe partially assembled NPCs in the nucleoplasm or cytoplasm of *MPS3-G816K* mutants that would suggest a defect in NPC insertion, perhaps due to the large number of NPCs present in the nuclear envelope [55] and the relatively short period of time in which the dominant mutant is expressed (Figure 3 and Figure 4). Because both SPBs and NPCs are observed in nuclear membrane areas that contain a single bilayer and there is no apparent defect in NPC insertion, we suspect that the SPB assembly defect associated with *MPS3-G186K* is the result of membrane composition rather than the excess membrane.

### *MPS3-G186K* mutants have defects in nuclear morphology

Membrane proliferation in *MPS3-G186K* mutants was also accompanied by an abnormal nuclear morphology. In many cases the nuclear membrane completely encircled regions of the cytoplasm, entrapping vesicles and other components (Figure 4A–4B). Membrane extensions and protrusions were observed in all *GAL-MPS3-G186K* cells (n = 50) and in 50% of *GAL-MPS3* cells (17 of 34), which do not over-proliferate the nuclear membrane (Figure 3B–3D). The remaining 50% of *GAL-MPS3* cells had a round or oval nuclear morphology similar to wild-type, depending on the cell cycle stage (Figure 3A).

**Figure 5:**
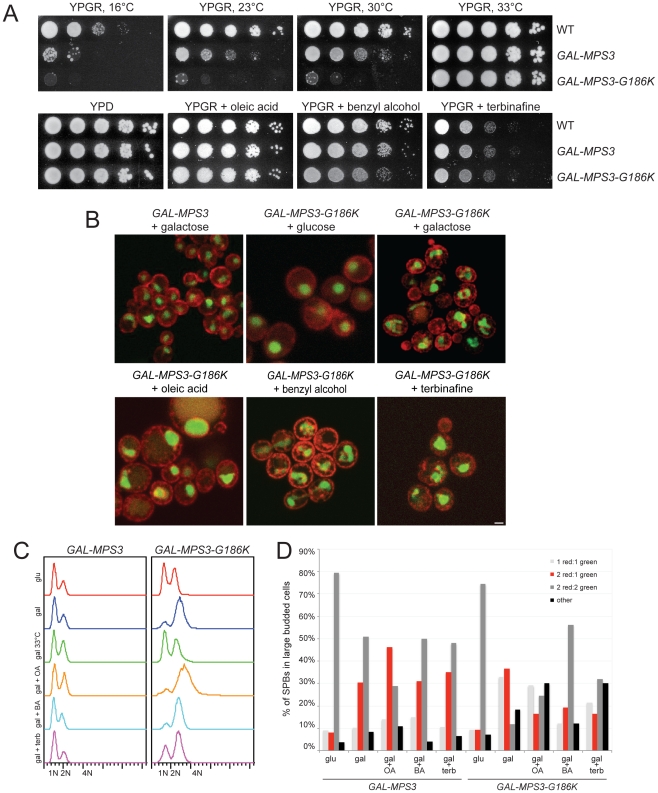
Alteration of membrane properties suppresses *MPS3-G186K*. (A) Wild-type (SLJ001), *GAL-MPS3* (SLJ995) and *GAL-MPS3-G186K* (SLJ1797) cells were tested for their ability to grow on YPGR, YPGR+0.2% benzyl alcohol, YPGR+5 mM oleic acid and YPGR+2.5 µg/ml terbinafine plates at 30°C in a serial dilution assay. Plates were grown for 2 d. Cells were also stamped to YPGR at 16°C for 7 d, 23°C for 3 d and 33°C for 2 d to test the effect of temperature on colony formation. (B) *GAL-MPS3* (SLJ4963) and *GAL-MPS3-G186K* (SLJ4965) containing HDEL-dsRed (red) and Pus1-GFP (green) were grown overnight in SC-LEU+2% raffinose then 2% glucose, 2% galactose, 2% galactose+5 mM oleic acid, 2% galactose+0.2% benzyl alcohol, or 2% galactose+1.25 µg/ml terbinafine was added for 6 h at 30°C to visualize the effects on nuclear morphology. Bar, 5 µm. (C–D) *GAL-MPS3* (SLJ4859) and *GAL-MPS3-G186K* (SLJ4864) cells were grown in SC-HIS+2% raffinose, then treated as in (B). A sample containing 2% galactose was also shifted to 33°C for 6 h. (C) DNA content was analyzed by flow cytometry. (D) In these same cells, the number of SPBs containing the indicated number of Spc42-mCherry and Spc110-GFP foci in large budded cells was quantitated (n>100 for all samples).

We were able to follow the formation of nuclear morphology defects in real-time in *GAL-MPS3-G186K* mutants and *GAL-MPS3* control cells using HDEL-dsRed to visualize the nuclear and ER membranes and Pus1-GFP to visualize the nucleus. Time-lapse image analysis showed that the nuclei in *MPS3* overexpressing cells underwent minor changes in nuclear shape—virtually all nuclei were round or oval shaped throughout the 3.5 h time-course, with a few extensions and protrusions forming in some nuclei only at later time points (Video S1; Figure 5B). In addition, we observed cells undergoing mitosis as the nucleus and nuclear membrane became elongated and then hour-glass shaped. In contrast, membrane extensions and nuclear deformation were easily observed in most *MPS3-G186K* cells by 1.5–2 h following addition of galactose (Video S2). At later time points, more severe membrane perturbation occurred in the mutant, often resulting in the formation of several masses of nuclear material within one cell (Figure 5B). Although some mutant nuclei elongated, none completed mitosis to form two distinct masses of DNA. Thus, it appears that membrane proliferation is linked to an abnormal nuclear morphology and inhibition of mitotic progression.

### Alteration of membrane composition alleviates the growth defect of *MPS3-G186K*

**Figure 6:**
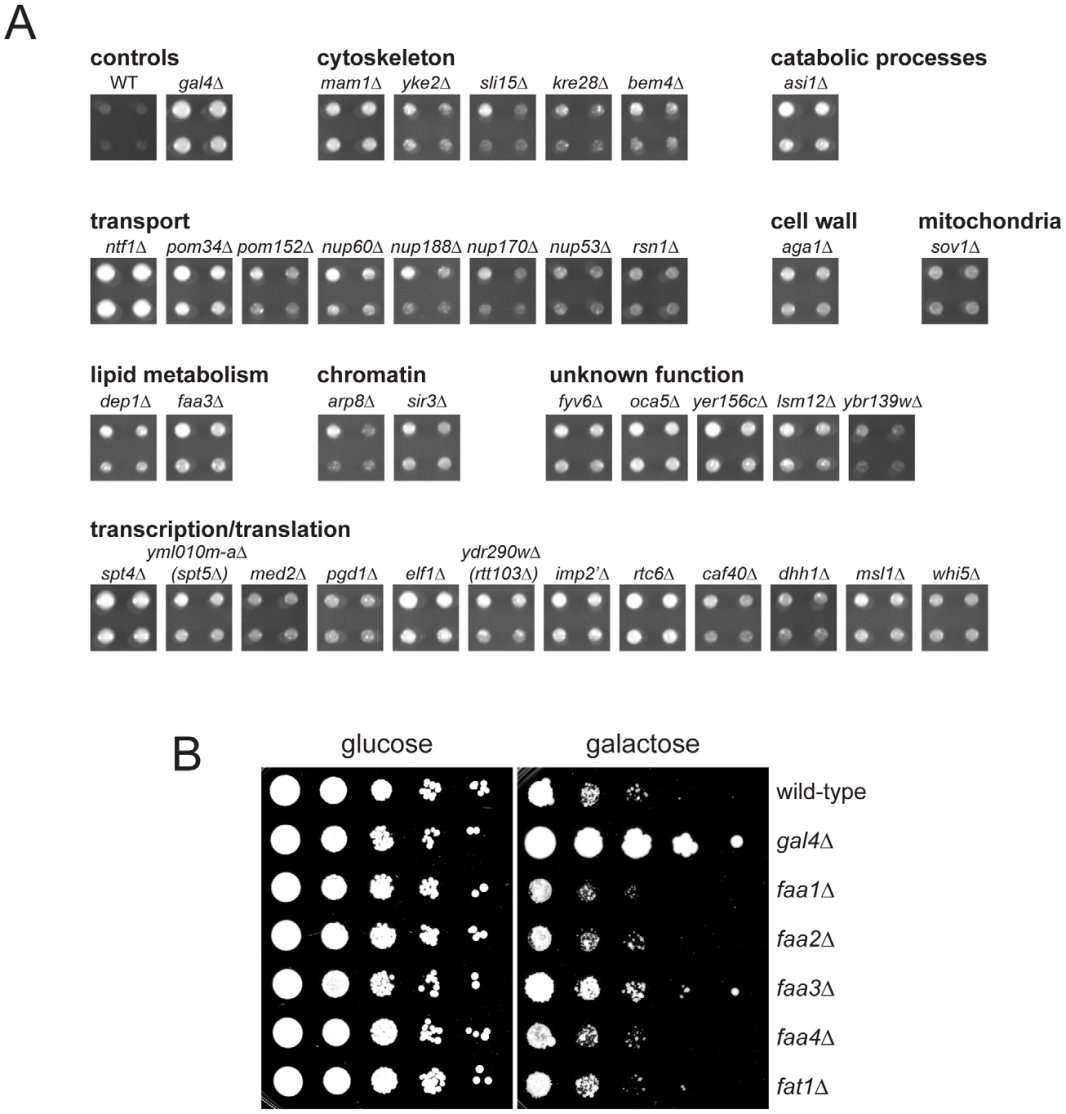
Suppressors of *MPS3-G186K* toxicity. (A) Mutants from the yeast deletion collection were transformed with p*URA3-GAL-MPS3-G186K*, and transformants were tested for their ability to grow on SC-URA+2% galactose at for 3 d at 30°C. Possible hits were then further examined by western blotting to ensure that Mps3-G186K was expressed at high levels, similar to the wild-type control. Shown here are the hits that both rescued the growth defect of *MPS3-G186K* and expressed wild-type levels of Mps3-G186K. A full list of genes recovered in this screen is presented in Table S2. Also shown are the wild-type strain and the *gal4Δ* mutant, which we isolated in the primary screen but eliminated in the secondary screen because it does not express *MPS3-G186K*. Hits are organized by function using GoSlim analysis. In some cases, we isolated a mutant that deletes part of an overlapping gene, which is indicated below in parenthesis. (B) The indicated genes were deleted in *GAL-MPS3-G186K* (SLJ1797) and growth was tested in a serial dilution assay at 30°C on YPD (glucose) for 2 d and YPGR (galactose) for 3 d.

To better understand the role that Mps3 plays in SPB insertion and membrane structure, we screened the yeast deletion collection for mutants that rescued the growth defect of *GAL-MPS3-G186K*. If SPB duplication defects in this mutant are the result of defects in membrane composition as our cytology suggests, then it should be possible to find mutants that alter the lipid composition of the nuclear membrane to compensate for *MPS3-G186K* expression. In total, 93 mutants were found to grow on SC-URA+2% galactose in the presence of p*URA3-GAL1-MPS3-G186K* (Table S2). Because a number of these genes likely affect transcription, translation, post-translational modification or galactose-induction of the dominant allele, we used western blotting as a secondary screen to identify mutants that expressed high levels of Mps3-G186K protein, similar to that observed in wild-type cells (data not shown). 37 mutants met this secondary criterion and are shown in Figure 6A together with a wild-type and *gal4Δ* control. *GAL4* encodes a transcription factor that is required for activation of genes in response to galactose, including expression from *GAL1* [56]. This mutant was identified in our primary screen, but as expected, failed to pass our secondary test for Mps3-G186K production and serves as a control (Table S2).

Of the 37 deletion mutants that restore growth to *MPS3-G186K*, a number of genes encode proteins involved in transcription or translation. While these do not affect production of Mps3-G186K, they most likely restore growth to the mutant by affecting expression of an unknown target. One possible candidate target is *POM34*, which has previously been shown to be regulated by translation and whose levels influence growth of mutants defective in SPB insertion, such as *mps2*, *bbp1* and *ndc1* [4], [25], [26]. Interestingly, we also found that deletion of *POM34*, *POM152* and several other nucleoporins suppressed *MPS3-G186K* mutants (Figure 6A). A number of these same deletion mutants were recently shown to rescue *mps3Δ* [26]. We proposed that deletion of the nucleoporins may rescue growth of cells lacking *MPS3* by blocking NPC assembly, thereby liberating a shared insertion factor involved in both SPB and NPC insertion. Alternatively, their deletion may rescue growth of *mps3Δ* cells by changing the physical properties of the nuclear membrane, such as membrane fluidity, to facilitate SPB duplication without Mps3. Our finding that *MPS3-G186K* mutants, which have defects in nuclear membrane structure and SPB duplication, are suppressed by the many of the same nucleoporin deletions as *mps3Δ* strongly suggests that changes in nuclear membrane properties, rather than liberation of an insertion factor, alleviate the growth defect in both cases. The fact that we isolated multiple nucleoporins as *MPS3-G186K* suppressors suggests a common mechanism of suppression. As we demonstrate below for *pom152Δ* and infer for the other nucleoporins, alteration of nuclear envelope properties appears to be responsible for suppression of *MPS3-G186K* as well as rescue of *MPS3* deletion.

In addition to nucleoporin deletions, we also found that deletion of two genes involved in lipid metabolism, *FAA3* and *DEP1*, rescues *MPS3-G186K* mutants. *DEP1* encodes a transcriptional regulator of many metabolic genes, including genes involved in phospholipid biosynthesis [57], [58]. *FAA3* encodes one of five acyl coA synthetases that catalyze the conversion of fatty acids into activated acyl-coA intermediates in the first step in phospholipid biosynthesis. Faa1, Faa2, Faa3, Faa4 and Fat1 localize to different subcellular compartments and display distinct specificities for medium, long and very-long chain fatty acids *in vitro* [59]. *FAA3* encodes a long chain or very long chain fatty acyl coA synthetase that is believed to be partially redundant with Fat1 *in vivo* [60]–[62]. However, we found that deletion of the other acyl coA synthetases, including *fat1Δ*, was unable to suppress the growth defect of *MPS3-G186K* (Figure 6B), suggesting that effects of Faa3 elimination are specific and that it may have a function in regulation of lipid composition at the nuclear membrane.

### Changes in lipid composition rescues growth, nuclear morphology, and SPB duplication defects of *MPS3-G186K*

Deletion or overexpression of *FAA3* leads to an alteration in cellular lipid content [60], [63], suggesting that changes in fatty acid levels or composition are what rescue growth of *MPS3-G186K* cells. To test this idea, we treated cells with chemicals that alter membrane composition or fluidity and examined the effects on cell growth and nuclear envelope morphology. We found that growth of *MPS3-G186K* mutants on YPGR was rescued by addition of the membrane fluidizer benzyl alcohol, the sterol biosynthesis inhibitor terbinafine and the fatty acid oleic acid (Figure 5A). In addition, increasing the temperature to 33°C, which increases membrane fluidity due to the cell's ability to adjust lipid composition by increasing levels of saturated and long chain fatty acids and altering the amount of sterols [64], [65], also suppressed the growth defect of *MPS3-G186K*, whereas decreasing the temperature to 23°C and 16°C, which decreases membrane fluidity, not only resulted in toxicity of *MPS3-G186K* but also exacerbated the phenotype associated with overexpression of wild-type *MPS3* (Figure 5A). Therefore, these data are consistent with the possibility that affecting lipid composition to increase membrane fluidity is sufficient to suppress *MPS3-G186K*. Our observation that overproduction of wild-type Mps3 is lethal at low temperatures suggests that *GAL-MPS3* cells may have an altered membrane composition due to a direct role of Mps3 in membrane homeostasis.

Further examination of cells by live cell imaging revealed that other defects associated with expression of *MPS3-G186K*, including abnormal nuclear morphology, cell division and SPB duplication, were at least partially suppressed by changing lipid composition. Oleic acid was particularly effective at suppressing defects in nuclear shape. The majority of nuclei appeared to maintain a round shape throughout a 3.5 h time course with few extensions or protrusions; however, few nuclei divided (Figure 5B). A rare example is shown in Video S3. In addition, analysis of DNA content by flow cytometry and examination of SPB insertion showed that most cells remained arrested in mitosis probably due to SPB duplication defects (Figure 5C–5D). It is unclear why oleic acid suppresses growth of *MPS3-G186K* on plates but does not rescue in liquid culture, although similar effects have been reported for numerous mutants (for example, [66], [67]).

Treatment of *MPS3-G186K* cells with other chemicals did not have as profound of an effect on nuclear morphology as oleic acid, perhaps because these drugs have pleiotropic effects on nuclear envelope structure (Figure 5B, Videos S4 and S5). For example, previous studies have shown that addition of benzyl alcohol affects NPC insertion [20], [21]. While it may alleviate membrane expansion caused by *MPS3-G186K*, benzyl alcohol addition might result in defects in NPC structure that lead to changes in nuclear morphology.

Nevertheless, despite terbinafine's partial rescue of nuclear shape, it eliminated the cell cycle arrest of *MPS3-G186K* and more than doubled the number of large-budded cells that contained two inserted SPBs (Figure 5C–5D). Although this could be due to a slow growth phenotype associated with terbinafine since no mitotic divisions were observed (Video S5 and Figure 5A), we favor the possibility that treatment with terbinafine affects sterol biosynthesis which in turn affects SPB duplication since we also observed that addition of other sterol inhibitors such as ketoconazole to media rescued growth of *MPS3-G186K* (Figure S4).

Treatment with benzyl alcohol also only partially rescued the nuclear shape and cell cycle arrest of *MPS3-G186K*, but it was able to restore a distribution of SPB intermediates similar to that of *GAL-MPS3* cells in galactose (Figure 5B–5D), suggesting that it alleviates the SPB duplication defect associated with the dominant mutant. Consistent with this idea, we observed cells undergoing chromosome segregation and cell division in the presence of benzyl alcohol (Video S4). Importantly, addition of benzyl alcohol had virtually no effect on cell cycle progression or SPB insertion in *GAL-MPS3* cells (Figure 5C–D). We also found that addition of chemicals had similar effects on *MPS3* and *MPS3-G186K* expression (Figure S5). Thus, the effects of *MPS3-G186K* on growth, nuclear morphology and SPB insertion can be rescued by altering the lipid composition of the cell using both genetic and chemical methods.

### *mps3-1* mutants have defects in lipid homeostasis

**Figure 7:**
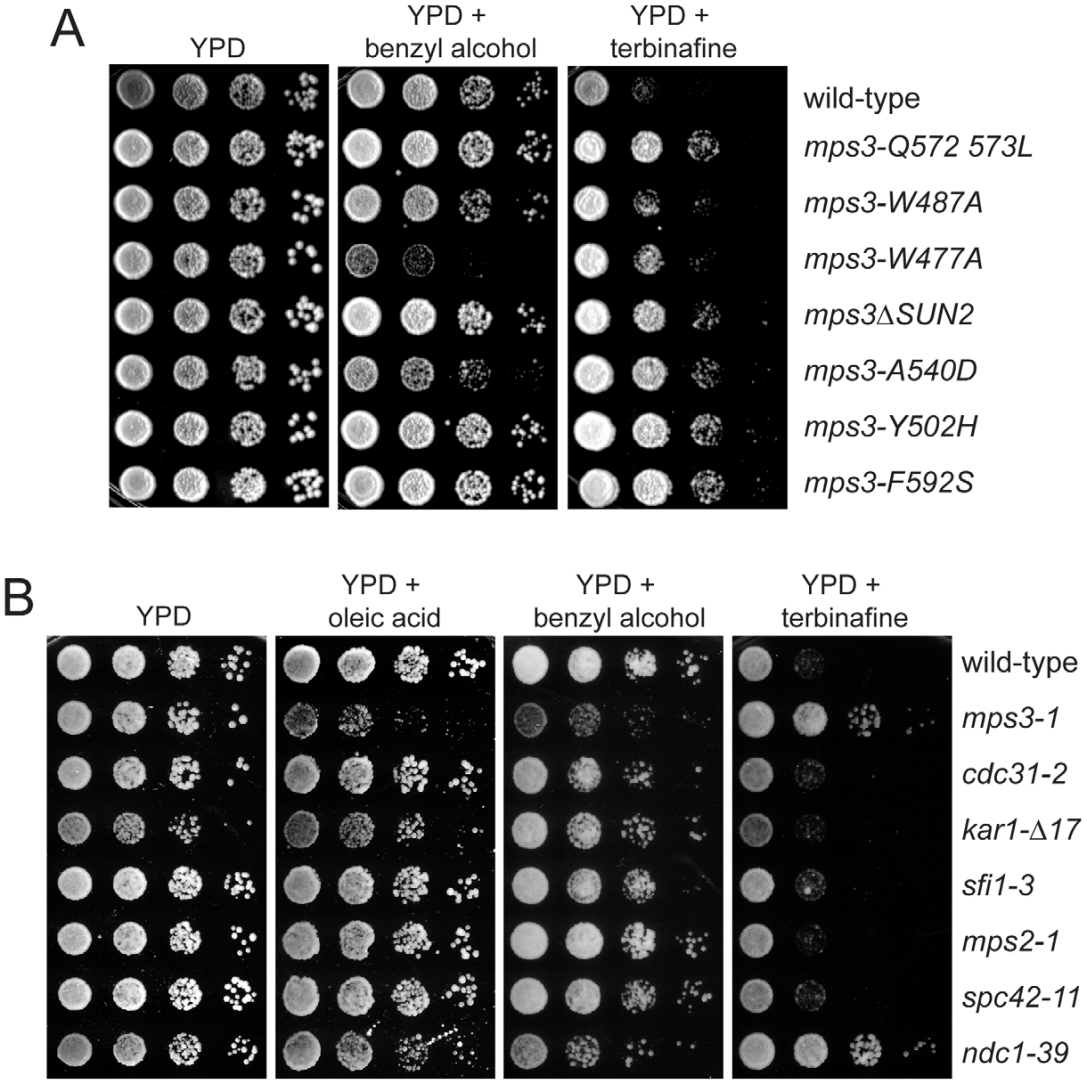
*mps3* mutants are sensitive to changes in membrane properties. (A) Wild-type (SLJ1779), *mps-Q572LQ573L* (SLJ1783), *mps3-W487A* (SLJ1785), *mps3-W477A* (SLJ1793), *mps3ΔSUN2* (SLJ1789), *mps3-A540D* (SLJ1787), *mps3-Y502H* (SLJ1781) and *mps3-F592S* (SLJ1791) cells, or (B) wild-type (SLJ001), *mps3-1* (SLJ910), *cdc31-2* (SLJ894), *kar1Δ17* (SLJ844), *sfi1-3* (SLJ1558), *mps2-1* (SLJ718), *spc42-11* (SLJ715) and *ndc1-39* (SLJ1740) cells, were grown to log-phase in YPD, then were tested for their ability to grown on YPD, YPD+0.2% benzyl alcohol and YPD+2.5 µg/ml terbinafine plates in a serial dilution assay at 23°C for 3 d. In (B) cells were also grown on YPD+5 mM oleic acid.

Analysis of our *mps3* deletion and point mutants showed that most are not sensitive to benzyl alcohol or sterol synthesis inhibitors (data not shown), suggesting that the synthetic effects we observe as a result of these treatments on *MPS3-G186K* mutants are due to a specific defect in Mps3 function that may be related to SPB insertion since *MPS3-G186K*, but not other mutants, display a SPB duplication defect (see Figure 1 and Figure 2). In support of this hypothesis, we found that virtually all mutants in the C-terminal SUN domain, which is required for SPB duplication [30]–[32], were resistant to terbinafine and found that a subset of these mutants, such as *mps3-1*, *mps3-A540D* and *mps3-W477A*, were also sensitive to benzyl alcohol (Figure 7A and 7B). Enhanced growth on terbinafine, which inhibits ergosterol biosynthesis [68], suggests that cells containing the *mps3-1*, *mps3-A540D* and *mps3-W477A* mutations have extremely fluid membranes; growth on membrane fluidizing agents like benzyl alcohol is toxic specifically to these mutants since they already have alterations in membrane composition. The more severe phenotype seen in some *mps3* alleles presumably reflects a greater alteration in the nuclear membrane, although it is possible that some of these mutants may have other defects as well [32], [40]. It is interesting that many, but not all, of the *mps3* mutants that are sensitive to benzyl alcohol are alleles that spontaneously diploidize. Perhaps the larger size of the nucleus in these mutants requires a greater need for lipid synthesis that cannot be met with two mutant copies of *MPS3*.

To determine if changes in lipid composition generally affect the SPB duplication process or are specific to *mps3* mutants, we compared the growth of different recessive mutants in SPB duplication on plates containing oleic acid, benzyl alcohol and terbinafine. Most SPB alleles tested, including mutants that disrupt early (*cdc31-2*, *kar1-Δ17*, *sfi1-3*), intermediate (*spc42-11*, *spc29-3*) and late steps (*mps2-1*, *bbp1-1*, *spc110-220*) in SPB duplication and SPB regulators (*mps1-1*, *tub4-1*) [3], grew at identical rates to wild-type under all conditions tested (Figure 7B; data not shown). The notable exception was the *ndc1-39* mutant, which is defective in both the late step of SPB duplication as well as in NPC assembly [5]. Like *mps3-1* mutants, *ndc1-39* cells exhibited increased sensitivity to oleic acid and benzyl alcohol and showed reduced sensitivity to terbinafine (Figure 7B). This suggests that membranes in *ndc1-39*, like *mps3-1* mutants, have very fluid membranes due to changes in membrane composition. While it is possible that the basis for the membrane defect in *ndc1-39* is related to its SPB insertion defect, we suspect that it is due instead to its role at the NPC since other SPB mutants, including *mps2-1* and *bbp1-1*, do not share this property. The fact that multiple *mps3* mutants, including alleles that are defective in early steps of SPB duplication and not known to cause defects in NPC assembly or function [30], [32], are specifically affected by growth on chemicals affecting lipid composition strongly supports our hypothesis that Mps3 function is intimately linked to membrane homeostasis.

### Cells lacking *MPS3* have aberrant lipid profiles

Our finding that *MPS3-G186K* mutants exhibit a severe membrane over-proliferation phenotype that is rescued by drugs and mutants that affect lipid synthesis, together with our observations that certain *mps3* mutants are sensitive to changes in membrane fluidity, indicates that Mps3 may play a role either directly or indirectly in regulating lipid composition in the cell. If this were the case, then we would anticipate that cells lacking *MPS3* would have an altered lipid composition compared to wild-type cells. To test this hypothesis, levels of phospholipids and neutral lipids were examined in whole cell preparations from five biological replicates of wild-type, *pom152Δ* and *pom152Δ mps3Δ* cells grown to mid-log phase in YPD at 30°C by electrospray ionization tandem mass spectrometry (ESI/MS/MS). *pom152Δ mps3Δ* mutants do not have an noticeable delay in cell division [26], so analysis of lipid composition in these cells will not be complicated by changes in temperature, growth media or cell cycle arrest that are associated with other *mps3* alleles.

**Figure 8:**
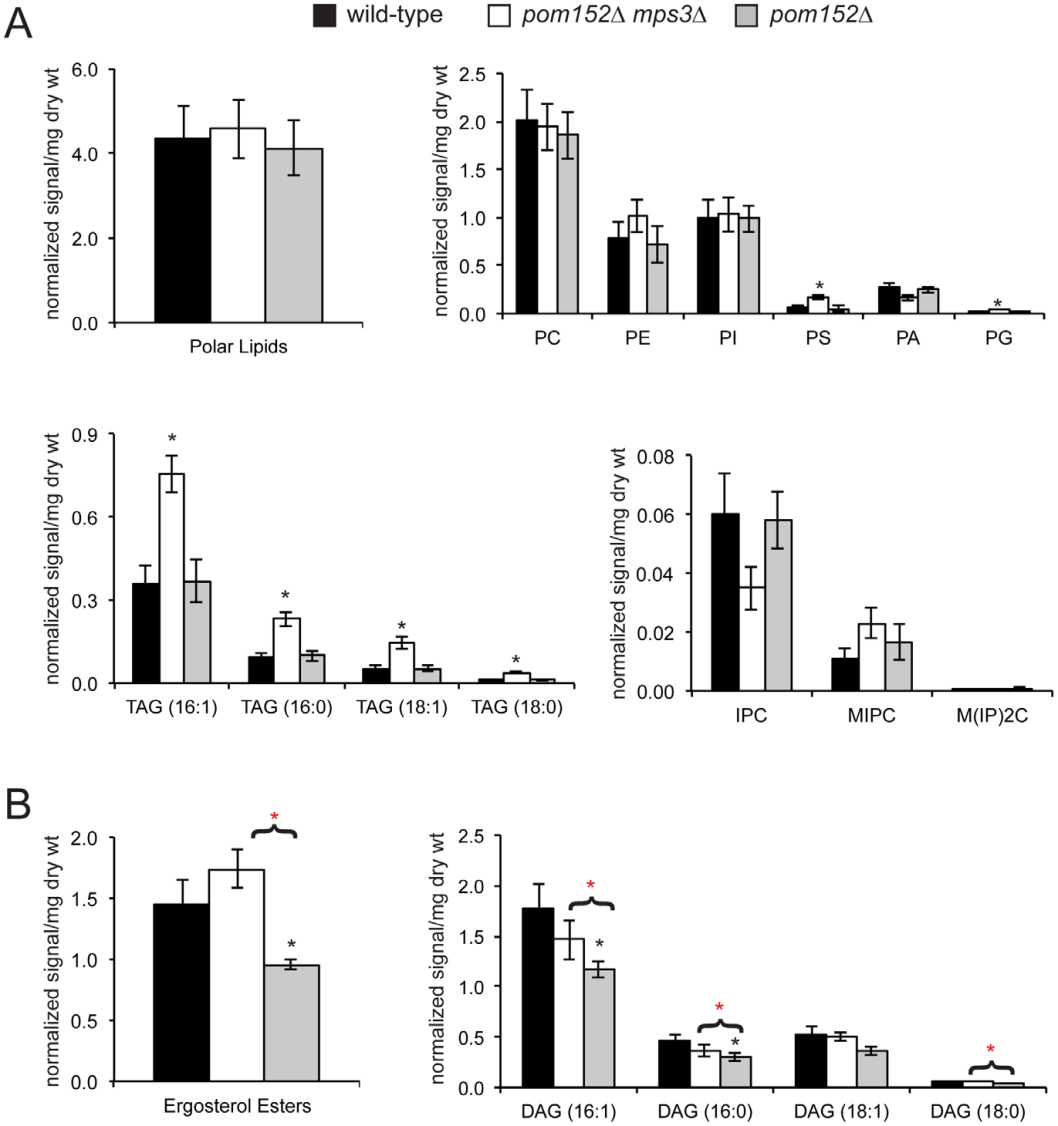
Aberrant lipid levels in cells lacking Mps3 function. Lipids were extracted from 5 independently grown mid-log phase cultures at 30°C of wild-type (SLJ001), *pom152Δ* (SLJ4260) and *pom152Δ mps3Δ* (SLJ4259) and analyzed by ESI/MS/MS. The total amount of each type of lipid was calculated per mg weight of wet yeast cells used, and average values for each class of lipid were tabulated along with the standard deviation from the mean. A complete summary of our lipidomics analysis is presented in Table S3. (A) Although there is no change in the levels of total polar lipids, different types of phospholipids are increased or decreased in *pom152Δ mps3Δ* (white) compared to wild-type (black) or *pom152Δ* (gray). *pom152Δ mps3Δ* cells also show increased amounts of TAG. (B) Cells lacking *POM152* only contain decreased levels of ergosterols and DAGs. Deletion of *MPS3* in these cells restores the level of ergosterols and DAGs to that observed in wild-type cells. (A & B) Black asterisks indicate values that are statistically significant from wild-type and red asterisks indicate values that are statistically significant from *pom152Δ* (p<0.05).

In cells lacking *MPS3*, we found that there was no statistically significant change in the overall levels of polar lipids compared to the controls (Figure 8A). However, examination of specific subclasses of phospholipids showed that *pom152Δ mps3Δ* mutants had 2.6 and 3.7 times the level of phosphatidyl serine (PS) and 2.0 and 2.3 times the level of phosphatidyl glycerol (PG) compared to wild-type and *pom152Δ* cells, respectively (Figure 8A; Table S3). Levels of phosphatidic acid (PA) and phosphatidyl ethanolamine (PE) were reduced 1.5 fold and increased 1.3 fold, respectively, in *pom152Δ mps3Δ* mutants compared with wild-type, although these values are not quite significant (p = 0.11 for PA, p = 0.37 for PE). The levels of other phospholipids such as phosphatidyl choline (PC) and phosphatidyl inositol (PI) were largely unchanged in wild-type, *pom152Δ* and *pom152Δ mps3Δ* samples. Thus, the distribution of polar lipids is affected in cells lacking Mps3 function.

Examination of neutral lipids revealed that cells lacking *MPS3* contained 2–3.3 times the level of triacylglycerols (TAGs) compared to wild-type and *pom152Δ* cells (Figure 8A; Table S3). These cells also had an altered distribution of the three yeast sphingolipids, including a 1.7 fold decrease in inositol phosphorylceramide (IPC) and a 2 fold increase in mannosylinositol phosphorylceramide (MIPC) compared to controls, although these changes were not quite statistically significant (p = 0.15 for IPC, p = 0.08 for MIPC). Even more importantly, we found that deletion of *POM152* alone resulted in an 1.5 fold decrease in ergosterol levels as well as a reduction in diacylglycerol (DAG), which was eliminated by simultaneous deletion of *MPS3* (Figure 8B; Table S3). This finding is critical for several reasons. First, it provides biochemical support for our theory that nucleoporin deletions result in changes in the physical properties of the nuclear membrane ([26] and above). Second, the fact that these changes in lipid composition are reversed by deletion of *MPS3* is compelling evidence that Mps3 directly affects membrane homeostasis. From our lipidomics data, we would predict that Mps3 likely acts at multiple steps in lipid biosynthesis, affecting both neutral lipid and phospholipid levels.

### Membrane homeostasis in cells lacking *MPS3* can be rescued by *FAA3*

**Figure 9:**
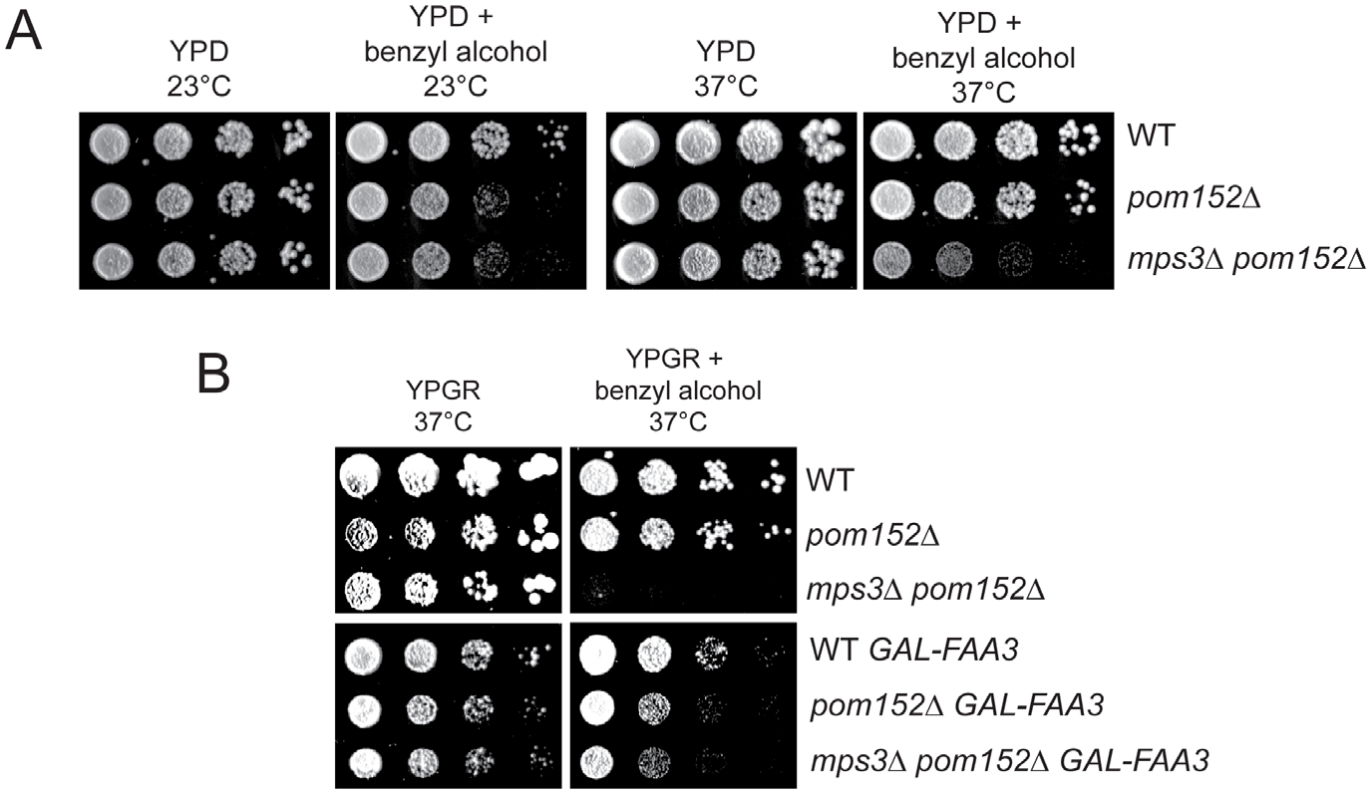
Membrane defects in cells lacking *MPS3* can be suppressed by *FAA3*. (A) Wild-type (SLJ001), *pom152Δ* (SLJ4260) and *pom152Δ mps3Δ* (SLJ5247) cells were tested for their ability to grow on YPD and YPD+0.2% benzyl alcohol at 23°C and 37°C in a serial dilution assay. Plates were grown for 2 d at 37°C and for 4 d at 23°C. (B) These same cells were transformed with an empty plasmid or with *GAL-FAA3* and tested for their ability to grow on YPGR and YPGR+0.2% benzyl alcohol at 37°C for 2 d in a serial dilution assay.

We would predict that changing properties of the nuclear membrane would result in growth defects in *pom152Δ mps3Δ* mutants if changes in lipid composition are required for SPB duplication and cell proliferation in the absence of *MPS3*. Indeed, we found that *pom152Δ mps3Δ* mutants were sensitive to benzyl alcohol, particularly at 37°C (Figure 9A). Although the lipid composition of *pom152Δ* mutants is altered, as was demonstrated in our lipidomics analysis (Figure 8; Table S3), growth of the single deletion is not affected by benzyl alcohol or other chemicals that affect membrane dynamics (Figure 9A). However, overexpression of *FAA3*, but not other acyl coA synthetases, partially rescued growth of *pom152Δ mps3*Δ mutants on benzyl alcohol-containing plates at high temperatures (Figure 9B; data not shown). Therefore, it seems that Faa3 is involved in *de novo* lipid synthesis required for nuclear membrane homeostasis, in particular, in membrane changes brought about by Mps3 that are essential for SPB insertion.

## Discussion

Our analysis of the functional domains of Mps3 has led to new insights into its role in SPB duplication and nuclear envelope homeostasis. The requirement for the transmembrane domain in Mps3 function extends previous data suggesting that Mps3 is a structural component of the half-bridge [30], [32]. However, our observation that multiple membrane domains can substitute for the Mps3 transmembrane domain is compelling evidence that no specific intra- or intermolecular interactions occur through this region of the protein, including transport through the NPC to the INM and anchorage at the SPB. We therefore tested the requirement for other conserved domains and were surprised to find that, at least when individually mutated, most were not essential for Mps3 function at the SPB in wild-type cells. Perhaps Mps3 has multiple binding partners and interaction domains due to the critical importance of SPB duplication in the maintenance of genomic integrity.

Through mutagenesis of the putative P-loop region of Mps3, we identified a new dominant allele that revealed a role for Mps3 in insertion of the newly duplicated SPB into the nuclear envelope. Although we could detect binding between Mps3 and ATP *in vitro* (data not shown) and glycine 186 is a conserved residue in the P-loop, we doubt that a defect in nucleotide binding is the underlying cause of the phenotypes we observed in *MPS3-G186K* mutants. This is because other mutants in conserved residues in the P-loop that also block ATP binding, such as S190A, G192K and S194A, did not result in defects in SPB duplication and nuclear envelope homeostasis. Also, alignment of Mps3 with other fungal orthologs shows that this motif is not conserved even within the *Saccharomyces* lineage. Thus, *MPS3-G186K* is likely a fortuitous antimorphic allele rather than a mutant that disrupts ATP binding *per se*.

Although overexpression of *MPS3* in our strains has few phenotypes at 30°C, its overproduction at 16°C and 23°C inhibits cell growth and leads to subtle changes in nuclear morphology (see Video S1). The simplest explanation for the temperature-dependent effect on cell viability when Mps3 is overproduced is that Mps3 plays a direct role in membrane homeostasis. Decreased amounts of saturated and long change fatty acids and changes in sterol levels, which accompany growth at lower at lower temperatures [64], [65], are incompatible with the alterations in membrane composition brought about by overproduction of Mps3. This incompatibility leads to a failure in SPB insertion as well as other changes in nuclear structure. Changes in lipid composition and nuclear architecture are exacerbated in the *MPS3-G186K* mutant such that this allele is toxic under most conditions, including growth at 30°C. Moreover, because the membrane pertubations are striking and growth arrest is tight, this allele is amenable to genetic and cell biological analysis.

Membrane over-proliferation in *GAL-MPS3-G186K* but not *GAL-MPS3* strongly suggests that it is not simply due to overproduction of an integral membrane protein, but rather are a unique consequence of overexpressing a mutant version of *MPS3*. We propose that this altered version of Mps3 titrates out key factors required for nuclear envelope homeostasis and/or SPB duplication. The most obvious candidate is the endogenous wild-type copy of Mps3 since SUN proteins are known to oligomerize and because a reduction of Mps3 at the spindle pole leads to defects in SPB duplication [23], [32], [69]–[71]. Consistent with this idea, mild overexpression of *MPS3* using a 2-micron plasmid partially rescues the growth defect of *GAL-MPS3-G186K* in cells grown at 30°C (Figure S2). Other candidates include the ribosome biogenesis factors Erb2 and Rrs1 that interact with Mps3 and are required for nuclear morphology [48].

Proliferation of intracellular membranes in response to increased levels of membrane proteins has been previously reported in a wide range of cell types; however, the intranuclear membranes that we observed following expression of *MPS3-G186K* are structurally distinct in several ways. First, unlike the flattened arrays of ER membrane known as karmallae formed following overexpression of *HMG1*, which encodes 3-hydroxy-3-methyl-glutaryl coenzyme A [72]–[75], the *MPS3-G186K*-dependent membranes appear to be exclusively intranuclear and most likely represent overproliferation of the INM. Second, unlike the intranuclear membranes formed upon overproduction of Nup53 that are devoid of NPCs [54], the *MPS3-G186K* membranes contain NPCs that are at least partially functional. In vertebrate cells, Sun1 localizes to NPCs and appears to play a role in their assembly [22]–[24], however, this function does not appear to be conserved in yeast despite the fact that highly curved membranes are involved in both NPC and SPB duplication since Mps3 does not co-localize with NPCs [48]. Third, the intranuclear arrays that form following *MPS3-G186K* expression are generally closely associated with the nuclear membrane, suggesting that proteins on the surface of the *MPS3-G186K*-induced membranes are able to interact with other nuclear envelope proteins. This is in contrast to intranuclear membrane arrays that form within the nucleoplasm and do not appear to associate with the nuclear membrane over large regions [76], [77]. Fourth, although the elaborate membrane extensions known as escapades formed by increasing levels of the peripheral membrane protein Esc1 also include NPCs [78], the excess membrane found in escapades originates at the nuclear vacuolar junction and results in predominant cytoplasmic extensions, whereas the *MPS3-G186K* membranes appear to begin as tubules inside of the nucleus that then fuse into intranuclear membranes. Fifth, unlike the membrane flares formed by disruption of proteins involved in the production of PA and DAG [53], [79]–[81], membrane proliferation in *MPS3-G186K* mutants was not restricted to the nucleolar region but rather appeared to occur at multiple sites inside of the nucleus. Interestingly though, the nucleolus was often partitioned away from the nucleus in a separate lobe in cells overexpressing both *MPS3-G186K* as well as *MPS3*, consistent with the idea that this region of the nuclear membrane has a unique molecular composition. In cells overexpressing *MPS3* or *MPS3-G186K*, we observed increased levels of most types of polar lipids as well as ergosterols compared to wild-type (data not shown). Levels of DAG were also elevated in *MPS3* overexpressing cells, but not in *MPS3-G186K* mutants, which might account for some of the differences we observed in membrane morphology and SPB duplication between these two strains.

In *MPS3-G186K* cells, the excess membrane appears to form as tubules within the nucleoplasm that then fuse to form the intranuclear membranes observed in the mutants. It is unclear how these tubules initiate, but they appear to be intermediates in nuclear envelope assembly. Curiously, similar tube-like structures have been detected in cells overexpressing *NUP53* and during the formation of nuclear membranes around sperm chromatin in *Xenopus* oocytes [54], [82]. Our observation that lipid composition affects cell viability, nuclear shape and membrane morphology of *MPS3-G186K* mutants suggests that specific properties of intranuclear tubules are critical for their formation, fusion and stacking to form an intact nuclear envelope. At least one important parameter is the amount of fatty acids produced by Faa3. This enzyme catalyzes acylation of long and very long chain fatty acids *in vitro*, which are important for the formation of highly curved membranes such as those that occur at sites of NPC and SPB insertion [83], [84]. By limiting the amount of these classes of fatty acids, it may be more difficult to form a membrane bend, which could help limit the membrane extension and protrusion driven by *MPS3-G186K*. We did not detect a difference in chain length in fatty acids in any of our *mps3* mutants, however (Table S3; data not shown). Possibly only a small pool of fatty acid side chains is modified making detection in a population assay difficult, or Faa3 may have a different substrate specificity *in vivo*. In addition, other membrane modifications such as sterol insertion may be a driving force in the control of nuclear envelope structure inside the cell. Many of the genes involved in sterol biosynthesis in yeast are encoded by essential genes [85] and thus, they are not present in the haploid yeast deletion collection so they would not have been discovered in our screen.

Deletion of NPC subunits suppresses the SPB duplication defect associated with mutation of several genes encoding SPB components [4], [25]–[27]. Our finding that deletion of *POM152* results in reduced levels of ergosterols and DAG compared to wild-type cells points to the possibility that the nucleoporin deletions affect the lipid composition of the nuclear envelope. This might occur through reduced transport of genes involved in lipid biosynthesis or through decreased export of the associated mRNAs. These changes in membrane composition could also account for genetic interactions between *pom152Δ* and genes involved in membrane fluidity, sterol synthesis, organelle integrity and membrane bending [18], [20], [21], [86].

In *pom152Δ mps3Δ* mutants, lipidomics analysis shows that the balance of ergosterol and DAG is reset to near wild-type levels, which could explain why SPB duplication and nuclear morphology defects were not observed in the double delete [26]. Other classes of lipids such as TAG are elevated in cells lacking *MPS3*, suggesting that Mps3 plays a direct role in regulation of membrane homeostasis. Our observation of allele specific defects in membrane proliferation and drug sensitivity in *mps3* mutants that was not detected in other SPB mutants further supports this hypothesis. The fact that these same alleles of *MPS3* are defective in SPB duplication indicates that Mps3-dependent changes in the nuclear envelope are important for formation of the new SPB, perhaps at multiple steps in SPB assembly. Studies in yeast, *Dictyostelium*, *C. elegans* and mammalian cells have shown that loss of SUN protein function leads to aberrant nuclear morphology [30], [32], [41], [48], [87]–[89]. Whether they are directly involved in the synthesis or maintenance of the nuclear membrane or are indirectly involved through binding to the nuclear lamina is not well understood [90]. Perhaps the best hint as to how SUN proteins might function in membrane homeostasis comes from two-hybrid analysis of the fission yeast SUN protein Sad1, which pointed to an interaction with the acetyl coA carboxylase enzyme Cut6 that is needed for *de novo* biosynthesis of long-chain fatty acids and mitotic progression [91], [92]. Much like the binding between SUN proteins and their ONM binding partners, known as KASH proteins, in the perinuclear space [35], we envision that SUN proteins like Mps3 may also associate with factors involved in membrane structure. Mps3 binding to enzymes or other proteins involved in membrane remodeling could be a mechanism to promote membrane bending and fusion at specific sites in the nuclear envelope, such as at the duplicating SPB or the NPC. However, Mps3 could also control membrane synthesis at the transcriptional level, by sequestering factors involved in lipid synthesis at the nuclear periphery. Future studies aimed at identification of Mps3 binding partners will help us better understand the role SUN proteins in membrane dynamics and nuclear morphology as well as elucidate the mechanism by which Mps3-dependent changes in the nuclear membrane enable duplicated SPBs to insert into the nuclear envelope.

## Materials and Methods

### Yeast strains and plasmids

All strains are derivatives of W303 (*ade2-1 trp1-1 leu2-3,112 ura3-1 his3-11,15 can1-100*) and are listed in Table S1 except those used in Figure 6 and Table S2, which are in BY4741 (*Mata his3Δ1 leu2Δ0 met15Δ0 ura3Δ0*) and were taken from the yeast deletion collection (Open Biosystems). Standard techniques were used for DNA and yeast manipulations.

Deletion of *MPS3*, fusion of *SPC110* and *NUP49* to GFP and fusion of *HTB2* (which encodes one of the two copies of histone 2B), *SPC42* or *NET1* to mCherry was done by PCR-based methods [93], [94]. Correct integration was confirmed by PCR.

The *MPS3* ORF and ∼500 bp of promoter were amplified by PCR and cloned into the XhoI and BamHI sites of a pRS306 vector containing GFP to construct pSJ650 (pRS306-*MPS3-GFP*) [41]. Construction of pSJ148 (pRS305-*MPS3*) has been previously described [32]. To overexpress *MPS3*, the entire open reading frame was inserted immediately adjacent to *GAL1* in pRS306 to create pSJ146 (pRS306-*GAL1-MPS3*). Plasmids were digested with BstEII or ApaI to target integration to *LEU2* or *URA3*, respectively. The number of copies of *MPS3* integrated was determined by Southern blotting. Deletion and point mutants, as well as transmembrane insertions, were generated in these plasmids using the Quick Change Mutagenesis Kit (Stratagene). Each deletion mutant contains an in-frame deletion of the indicated amino acids; in the case of 2xpQ mutant, an extra copy of the pQ domain was inserted immediately after the pQ coding sequence. Sequencing was performed to confirm correct base pair substitutions or deletions were made.

To test for rescue of *MPS3-G186K*, plasmids containing the indicated SPB genes were taken from the yeast tiling library [95] and transformed into SLJ1797. For dilution assays, 5 OD_600_ of cells were serially-diluted 10-fold in sterile growth media and stamped onto agar plates. YPD has 2% glucose and YPGR has 2% galactose and 2% raffinose as the carbon source. Chemicals were purchased from Sigma and were added to media in the following amounts: 5 mM oleic acid, 0.2% benzyl alcohol, 1.25 µg/ml terbinafine and 1 µg/ml ketoconazole.

### Cytological techniques

Localization of Mps3-GFP, H2B-mCherry, the nuclear localization sequence (NLS) reporter, HDEL-dsRed and Pus1-GFP were visualized as previously described [41]. Briefly, 1 ml of culture was centrifuged, washed with 1 ml ddH_2_O, resuspended in approximately 100 µL of ddH_2_O and 10 µL was placed on a 25% gelatin pad for image analysis. Fluorescence was performed using a confocal microscope (LSM-510-META; Zeiss) equipped with a ConforCor 3 module with avalanche photodiode detectors, which allow single photon counting, with a 100× 1.46 NA α-Plan Fluar objective (Zeiss). GFP was excited using a 488-nm Argon laser line, while mCherry was excited with a 561-nm HeNe laser line with the appropriate filter sets. Emitted photons were collected through BP 505–540 nm and LP 580 nm filters, with a pinhole size of 1.03 Airy units according to the green channel. Data was acquired using AIM v.4.2 software (Zeiss, Inc.). Images were collected with 8–10 image stacks with a 0.3 micron step size through the cells at room temperature. Images were processed using ImageJ software (NIH). At least two independent transformants of each genotype were analyzed by fluorescence microscopy in at least three independent experiments. N∶C ratios were calculated as previously described [28].

Time-lapse image analysis of *GAL-MPS3* or *GAL-MPS3-G186K* strains was performed on cultures grown overnight in YEP plus 2% raffinose at 30°C then diluted back to an OD_600_ of 0.2 and grown in the same media for 2 h. 2% galactose and nothing, 0.2% benzyl alcohol, 5 mM oleic acid, 1.25 µg/ml terbinafine or 1 µg/ml ketoconazole were added at 30°C for 30 min. One ml of culture was centrifuged to concentrate the cells and 5 µL was placed on a 1% agarose pad containing the same media and chemical treatment. Fluorescent images were taken from 1 h to 3 h 30 min after galactose induction using the same microscope configuration but with a 40× 1.3 NA oil objective (Zeiss). Emitted photons were collected through BP 505–540 nm and LP 580 nm filters, with a pinhole size of 1.03 Airy units according to the green channel. Images were collected with 17 image stacks with a 0.7 micron step size through the cells at room temperature. The 512×512 images were binned spatially 2×2 and a max projection was applied for each Z-slice. All data processing and video generation were done in ImageJ (NIH).

For the characterization of the SPB duplication using the red/green foci assay, images were acquired with a 100× 1.4 NA oil objective on an inverted Zeiss 200 m equipped with a Yokagawa CSU-10 spinning disc. 488 nm excitation and 568 nm excitation were used for GFP and mCherry, respectively, and emission was collected through BP 500–550 nm and BP 590–650 nm filters, respectively, onto a Hamamatsu EMCCD (C9000-13). For each channel, a Z-stack was acquired using 0.6 or 0.7 micron spacing. 13 total slices were acquired, and a max projection image was created for analysis of foci using ImageJ (NIH).

Analysis of DNA content by flow cytometry, EM, and protein localization by indirect immunofluorescence microscopy were performed as previously described [30], [32], [96]. Poly(A)+ *in situ* hybridization was performed using an Cy3-labeled oligo(dT)50 probe as described [97]. Cells were examined with a Zeiss Axioimager using a 100× Zeiss Plan-Fluar lens (NA = 1.45), and images were captured with a Hamamatsu Orca-ER digital camera and processed using ImageJ (NIH).

### Western blotting

The following primary antibody dilutions were used: 1∶1000 anti-Flag (Sigma) and 1∶1000 anti-glucose-6-phosphate dehydrogenase (G6PDH; Sigma). Fluorescently conjugated secondary antibodies were used at 1∶10000 for analysis and quantification using the Odyssey system (Li-Cor).

### ESI-MS/MS lipid profiling

Yeast cells were grown overnight at 30°C in YPD, diluted to OD_600_ of 0.2 in the same media and allowed to grow for 6–8 h at 30°C. The wet weight of the sample was recorded and lipids were extracted by the following procedure. Pelleted cells were homogenized using glass beads and water and the aqueous homogenized cells were transferred to 15 ml Teflon-lined glass tubes. To 0.8 parts (1.6 ml) of homogenized cells in aqueous solution, 1 part (2 ml) of chloroform and 2 parts (4 ml) of methanol were added. The slurry was shaken, 1 part (2 ml) of chloroform and 1 part of water (2 ml) were added. After shaking well, the slurry was centrifuged at low speed for 5–10 min and the lower layer was removed. One part of chloroform was added, shaken, centrifuged and the lower layer was again removed. This was repeated, and the combined lower layers were washed once with a small volume of 1 M KCl and once with a small volume of water. The combined and washed chloroform extracts were dried under liquid nitrogen and stored at −20°C in 2 ml Teflon-lined glass vials.

An automated electrospray ionization-tandem mass spectrometry (ESI-MS/MS) approach was used to analyze lipid composition in these samples, and data acquisition and analysis were carried out as described previously [98] with modifications. A 5 to 20 µl of aliquot of extract in chloroform was used from each 1 ml lipid sample. Precise amounts of internal standards, obtained and quantified as previously described [99], were added in the following quantities: 0.3 nmol di12:0-PC, 0.3 nmol di24:1-PC, 0.3 nmol 13:0-lysoPC, 0.3 nmol 19:0-lysoPC, 0.3 nmol di12:0-PE, 0.3 nmol di23:0-PE, 0.3 nmol 14:0-lysoPE, 0.3 nmol 18:0-lysoPE, 0.3 nmol di14:0-PG, 0.3 nmol di20:0(phytanoyl)-PG, 0.3 nmol di14:0-PA, 0.3 nmol di20:0(phytanoyl)-PA, 0.2 nmol di14:0-PS, 0.2 nmol di20:0(phytanoyl)-PS, 0.46 nmol 16:0-18:0-PI, 0.33 nmol di18:0-PI, 3.1 nmol tri17:1 TAG, and 4.6 nmol di15:0 DAG. The sample and internal standard mixture was combined with solvents, such that the ratio of chloroform/methanol/300 mM ammonium acetate in water was 300/665/35, and the final volume was 1.4 ml.

Unfractionated lipid extracts were introduced by continuous infusion into the ESI source on a triple quadrupole MS (4000QTrap, Applied Biosystems). Samples were introduced using an autosampler (LC Mini PAL, CTC Analytics AG, Zwingen, Switzerland) fitted with the required injection loop for the acquisition time and presented to the ESI needle at 30 µl/min.

Sequential precursor and neutral loss scans of the extracts produce a series of spectra with each spectrum revealing a set of lipid species containing a common head group fragment. Lipid species were detected with the following scans: PC and lysoPC, [M+H]^+^ ions in positive ion mode with Precursor of 184.1 (Pre 184.1); PE and lysoPE, [M+H]^+^ ions in positive ion mode with Neutral Loss (NL) of 141.0 (NL 141.0); PG, [M+NH_4_]^+^ in positive ion mode with NL 189.0 for PG; PI, [M+NH_4_]^+^ in positive ion mode with NL 277.0; PS, [M+H]^+^ in positive ion mode with NL 185.0; PA, [M+NH_4_]^+^ in positive ion mode with NL 115.0; IPC, [M−H]^−^ in negative ion mode with Precursor of 259.0; MIPC, [M−H]^−^ in negative ion mode with Precursor of 421.0; M(IP)_2_C, [M−H]^−^ in negative ion mode with Precursor of 663.1; (lysoPG, [M−H]− in negative ion mode with Precursor of 152.9 used as a standard for IPC, MIPC, M(IP)_2_C). The collision gas pressure was set at 2 (arbitrary units). The collision energies, with nitrogen in the collision cell, were +28 V for PE, +40 V for PC, +25 V for PA, PI and PS, +20 V for PG, −72 for IPC, −80 for MIPC, −75 for M(IP)_2_C, −57 for lysoPG. Declustering potentials were +100 V for all positive mode scans, −180 for IPC, MIPC, and M(IP)_2_C, −100 for lysoPG. Entrance potentials were +15 V for PE and +14 V for PC, PA, PG, PI, and PS, −13 for IPC, MIPC, and M(IP)_2_C, −10 for lysoPG. Exit potentials were +11 V for PE and +14 V for PC, PA, PG, PI, PS, −10 for IPC, MIPC, and M(IP)_2_C, −14 for lysoPG. The scan speed was 50 or 100 u per sec. The mass analyzers were adjusted to a resolution of 0.7 u full width at half height. For each spectrum, 9 to 150 continuum scans were averaged in multiple channel analyzer (MCA) mode. A series of neutral loss scans were used to detect ergosterol esters, DAG, and TAG species as [M+NH_4_]^+^ ions. The scans targeted losses of various fatty acids as neutral ammoniated fragments: NL 285.2 (17∶1, for the TAG internal standard); NL 259.2 (15∶0, for the DAG internal standard); NL 271.2 (16∶1); NL 273.2 (16∶0); NL 299.2 (18∶1); and NL 301.2 (18∶0). The scan speed was 100 u per sec. The collision energy was +25 V, entrance potential was +14 V, and exit potential was +14 V. Fifty continuum scans were averaged in MCA mode. The source temperature (heated nebulizer) was 100°C, the interface heater was on, +5.5 kV was applied to the electrospray capillary, the curtain gas was set at 20 (arbitrary units), and the two ion source gases were set at 45 (arbitrary units).

The background of each spectrum was subtracted, the data were smoothed, and peak areas integrated using a custom script and Applied Biosystems Analyst software, and the data were corrected for overlap of isotopic variants (A+2 peaks). The first and typically every 11^th^ set of mass spectra were acquired on the internal standard mixture only. Peaks corresponding to the target lipids in these spectra were identified and molar amounts calculated in comparison to the two internal standards on the same lipid class. To correct for chemical or instrumental noise in the samples, the molar amount of each lipid metabolite detected in the “internal standards only” spectra was subtracted from the molar amount of each metabolite calculated in each set of sample spectra. The data from each “internal standards only” set of spectra was used to correct the data from the following 10 samples. Finally, the data were corrected for the fraction of the sample analyzed and normalized to the mg wet weight to produce data in the units nmol/mg, except DAG, TAG, and ergosterol esters. For analysis of these classes, there is variation in ionization efficiency [100]. Thus, the ratio of the MS response of the internal standard does not provide a value that is directly proportional to the content of each species. DAG, TAG, and ergosterol ester amounts are thus expressed as relative mass spectral signal/mg wet weight, where a signal of 1.0 represents a signal equal to the signal of 1 nmol tri17:1 TAG, or 1 nmol di15:0 DAG (the internal standards).

## Supporting Information

Figure S1 A specific transmembrane domain sequence is not required for Mps3 localization or function at the SPB. (A) Transmembrane regions from other integral membrane proteins were used to replace that of Mps3, and these proteins were localized as in Figure 1C. In the case of mps3ΔTM(Heh1TM2)-GFP (SLJ4320), imaging was performed with the covering plasmid since this allele is non-functional. Bar, 2 µm. (B) Sequence of Mps3 transmembrane domain and the transmembrane regions from other membrane proteins that were used to replace that of Mps3. Summarized is the ability of each chimeric construct to complement *mps3Δ* (SLJ2694), which was scored based on growth on 5-FBA at 30°C. ++, indicates robust growth equal to wild-type *MPS3*; +, indicates good growth with colony sizes smaller than wild-type and no increase in ploidy; +/−, indicates poor growth and/or increase in ploidy; −, indicates no growth similar to an empty vector. In addition, the localization of each chimeric protein was determined as in (A). Over 30 cells of each genotype were examined and the predominant localization pattern is summarized. Those marked with an asterisk contained the covering plasmid. Also shown is the predicted topology of Mps3 and the chimeric proteins based on TMpred [102]: here, i-o means inside to outside while o-i means the opposite side in. In some cases topology prediction was ambiguous. In most cases, there was a strong prediction that the chimeric protein would have the i-o topology resembling Mps3, indicating that failure of the chimeric protein to complement is not necessarily due to reversal of membrane orientation.
(EPS)

Figure S2 Suppression of *MPS3-G186K* toxicity by overexpression of SPB components. *GAL-MPS3-G186K* (SLJ1797) was transformed with the indicated genes on a 2μ-*LEU2* plasmid from the yeast tiling library [95] and growth was examined in a serial dilution assay. Cells grown on SC-URA-LEU+2% glucose were incubated for 3 d at 30°C while cells grown on SC-URA-LEU+2% galactose+2% raffinose were incubated for 5 d at 30°C.
(EPS)

Figure S3 Nuclear import and export is significantly affected in *MPS3-G186K*. (A–B) Wild-type (SLJ5384), *GAL-MPS3* (SLJ5093) and *GAL-MPS3-G186K* (SLJ5094) strains containing H2B-mCherry and cNLS-GFP were grown in SC-LEU+2% raffinose overnight, then cultures were divided: to one set, 2% glucose was added and to a second set, 2% galactose was added, and cultures were incubated for 2 h at 30°C. The distribution of cNLS-GFP in the nucleus versus cytoplasm was analyzed by live cell imaging (A) followed by quantitation of the nuclear to cytoplasmic ratio (N∶C) of cNLS-GFP protein (B). (C–D) *GAL-MPS3* (SLJ995) and *GAL-MPS3-G186K* (SLJ1797) strains were also grown in YEP+2% raffinose overnight, then cultures were divided: to one set, 2% glucose was added and to a second set, 2% galactose was added, and cultures were incubated for 4 h at 30°C. Cells were harvested and polyA localization was analyzed by fluorescent in situ hybridization using Cy3-labelled oligo-dT (red). DNA was stained using DAPI (blue) (C). The distribution of mRNA in the nucleus vs. cytoplasm was quantitated to generate a N∶C ratio (D). n>150 for all samples; statistical significance compared to the glucose sample is shown. Bar, 5 µm.
(EPS)

Figure S4 Sterol biosynthesis inhibitors suppress the growth defect of *MPS3-G186K*. (A) Wild-type (SLJ001), *GAL-MPS3* (SLJ995) and *GAL-MPS3-G186K* (SLJ1797) cells were tested for their ability to grow on YPD, YPGR and YPGR+1 µg/ml ketoconazole plates at 30°C in a serial dilution assay. Plates were grown for 2 d. (B) In addition, *GAL-MPS3-G186K* (SLJ4965) containing HDEL-dsRed (red) and Pus1-GFP (green) grown overnight in SC-LEU+2% raffinose was analyzed at 6 h at 30°C following the addition of 2% galactose+1 µg/ml ketoconazole to determine the effects on nuclear morphology. Bar, 5 µm. (C) DNA content was also analyzed by flow cytometry.
(EPS)

Figure S5 Levels of Mps3 and Mps3-G186K following treatment with oleic acid, terbinafine and benzyl alcohol. Quantitative western blotting to examine the levels of Mps3-3xFLAG (SLJ5138) and Mps3-G186K-3xFLAG (SLJ5140) following treatment with different chemicals for 6 h in YPGR or following a shift to 33°C for 6 h in YPGR (as in Figure 5). Blots were probed with anti-FLAG antibodies to detect the level of Mps3 or Mps3-G186K protein and with anti-G6PDH, which serves as a loading control. Cells grown in glucose and galactose were assigned values of 0 and 1, respectively, and levels in treated cells in a representative experiment are shown.
(EPS)

Table S1 Yeast strains used in this study.
(XLS)

Table S2 Suppressors of *GAL-MPS3-G186K*.
(XLS)

Table S3 Lipidomics analysis of cells lacking *MPS3*.
(XLS)

Video S1 Growth of *GAL-MPS3* in YPGR. HDEL-dsRed marks the nuclear membrane and ER while Pus1-GFP highlights the nucleus in cells containing *GAL-MPS3* grown in YEP plus 2% raffinose and 2% galactose at 23°C. From ∼75–90 min, the upper left cell undergoes mitosis, where the nucleus is observed to form an hour-glass shape and then elongate between the mother and daughter cell. Following the completion of mitosis, the nucleus becomes round again. Another mitosis was observed in the cell in the bottom right between ∼130–160 min. At later time points, a few changes in nuclear morphology were observed but overall, the nuclei largely retained a relatively normal shape throughout the 3.5 h time course. Bar, 5 µm.
(MOV)

Video S2 Growth of *GAL-MPS3-G186K* in YPGR. HDEL-dsRed marks the nuclear membrane and ER while Pus1-GFP highlights the nucleus in cells containing *GAL-MPS3-G186K* grown in YEP plus 2% raffinose and 2% galactose at 23°C. At the beginning of the time course, the second cell from the upper right assumes an oval shape, suggesting it is starting mitosis. The nucleus elongates and two masses separate into the mother and daughter cell, but Pus1-GFP signal can still be detected in a thin region between the two nuclei throughout the entire time series. Around 150 min, deformations of the nuclei start to become apparent; in some cases such as the upper right cell, regions of the nucleus appear to form lobes, which can then bud from and fuse with each other. Bar, 5 µm.
(MOV)

Video S3 Growth of *GAL-MPS3-G186K* in YPGR plus 5 mM oleic acid. HDEL-dsRed marks the nuclear membrane and ER while Pus1-GFP highlights the nucleus in cells containing *GAL-MPS3-G186K* grown in YEP plus 2% raffinose and 2% galactose at 23°C. In addition, 5 mM oleic acid was added, which suppresses most of the dynamic changes in nuclear shape. A cell in the bottom center enters mitosis at ∼195 min. Bar, 5 µm.
(MOV)

Video S4 Growth of *GAL-MPS3-G186K* in YPGR plus 2% benzyl alcohol. HDEL-dsRed marks the nuclear membrane and ER while Pus1-GFP highlights the nucleus in cells containing *GAL-MPS3-G186K* grown in YEP plus 2% raffinose and 2% galactose at 23°C. In addition, 2% benzyl alcohol was added. Although the nuclear morphology in these cells is somewhat abnormal, three mitotic divisions were observed during the time-lapse, indicating that the addition of benzyl alcohol suppressed the metaphase arrest associated with expression of the dominant mutant. Bar, 5 µm.
(MOV)

Video S5 Growth of *GAL-MPS3-G186K* in YPGR plus 1.25 µg/ml terbinafine. HDEL-dsRed marks the nuclear membrane and ER while Pus1-GFP highlights the nucleus in cells containing *GAL-MPS3-G186K* grown in YEP plus 2% raffinose and 2% galactose at 23°C. In addition, 1.25 µg/ml terbinafine was added. Although some nuclei elongate as if entering mitosis (upper center cell), no nuclear division was observed during the time course or in subsequent image analysis. The nuclei also appeared to contain several extensions, similar to cells lacking terbinafine. Bar, 5 µm.
(MOV)
